# Enhanced immunogenicity and dose-sparing efficacy of self-amplifying RNA vaccines against seasonal influenza across subtypes

**DOI:** 10.1080/22221751.2026.2668752

**Published:** 2026-05-04

**Authors:** Mengting Huang, Yun Quan, Ruyi Chen, Hao Gu, Wenjun Song, Feng Zhu, Simin Feng, Jinzhong Lin, Jing Lu, Xuechun Li, Wansheng Li, Qianyu Pan, Hongli Li, Lei Sun, Tao Jiang, Bihui Zhang, Xinqian Yin, Dandan Wei, Changrong Yang, Donglan Liu, Zhongfang Wang, Weixin Jia, Jincun Zhao, Jieshi Yu, Xiaobo Li, Xuedong Yu, Xiaojing Yue, Chufang Li, Fan Bu, Zhichao Miao, Qiong Zhang

**Affiliations:** a State Key Laboratory of Respiratory Disease, National Clinical Research Center for Respiratory Disease, Guangzhou Institute of Respiratory Health, The First Affiliated Hospital of Guangzhou Medical University, Guangzhou, People’s Republic of China; b Guangzhou National Laboratory, Guangzhou, People’s Republic of China; c Key Laboratory of Virology and Biosafety, Wuhan Institute of Virology, Chinese Academy of Sciences, Wuhan, People’s Republic of China; d Hubei Jiangxia Laboratory, Wuhan, People’s Republic of China; e College of Life Sciences, University of Chinese Academy of Sciences, Beijing, People's Republic of China; f State Key Laboratory of Genetic Engineering, School of Life Sciences, Zhongshan Hospital, Fudan University, Shanghai, People’s Republic of China; g Shanghai Institute of Infectious Disease and Biosecurity, Fudan University, Shanghai, People’s Republic of China; h Zhangjiang mRNA Innovation and Translation Center, Fudan University, Shanghai, People’s Republic of China; i Shanghai RNACure Biopharma Co., Ltd., Shanghai, People’s Republic of China; j State Key Laboratory of Pathogen and Biosecurity, Academy of Military Medical Sciences, Beijing, People’s Republic of China; k National Avian Influenza Para-Reference Laboratory (Guangzhou), Guangdong Engineering Laboratory for Medicament of Zoonosis Prevention and Control, College of Veterinary Medicine, South China Agricultural University, Guangzhou, People’s Republic of China; l State Key Laboratory of Swine and Poultry Breeding Industry, Agro-Biological Gene Research Center, Guangdong Academy of Agricultural Sciences, Guangzhou, People’s Republic of China

**Keywords:** Self-amplifying RNA, influenza B virus, dose-sparing, seasonal influenza, trivalent vaccine

## Abstract

Recent clinical data on seasonal influenza mRNA vaccines have demonstrated suboptimal efficacy against the influenza B virus (IBV). We employed sequence optimization strategies that successfully enhanced the antigen expression of hemagglutinin (HA) and developed mRNA vaccine candidates targeting the WHO-recommended strains. When administered at a low dose (0.1 μg), both mono-and trivalent influenza A mRNA vaccines induced robust humoral immunity and conferred complete protection against homologous viral challenge in murine models, outperforming the quadrivalent inactivated vaccine (QIV, 2 μg). In contrast, IBV mRNA vaccines at an equivalent dose failed to elicit detectable antibodies and offered no protection, consistent with prior evidence of suboptimal immunogenicity in human trials. These findings highlight strain-specific immunogenicity constraints inherent to conventional mRNA platforms. To overcome these limitations, we systematically compared three distinct RNA vaccine modalities: (1) nucleoside-modified mRNA, (2) self-amplifying RNA (saRNA), and (3) circular RNA (circRNA). Notably, a single 0.1 µg dose of the trivalent saRNA vaccine elicited robust humoral immunity and provided complete protection against IBV challenge, whereas mRNA vaccination achieved only 14% survival. Importantly, long-term antibody monitoring over 20 weeks showed that saRNA at the low 0.1 μg dose maintained high antibody levels, with a markedly more durable response to IBV antigens than those of other platforms. Moreover, the trivalent mRNA vaccine exhibited a favourable safety profile, with no obvious abnormal body weight changes or serum biochemical abnormalities observed after immunization. Our findings advocate for strain-adaptive platform selection: conventional mRNA for generating rapid, high-magnitude responses against influenza A and next-generation saRNA vaccines for enhanced dose efficiency, particularly against IBV.

## Introduction

Influenza B virus (IBV), despite being historically overshadowed by influenza A virus (IAV) in pandemic potential, accounts for a substantial proportion of seasonal influenza cases (30–87% annually) and imposes severe disease burdens, particularly in pediatric and elderly populations [1,2]. Unlike influenza A, which undergoes frequent antigenic shifts, IBV evolves primarily through drift in its hemagglutinin (HA) and neuraminidase (NA) proteins, leading to mismatches between vaccine strains and circulating viruses [3].

Current influenza vaccines-primarily egg-based inactivated or recombinant HA vaccines-suffer from critical limitations: (1) production timelines of 6–8 months, often resulting in suboptimal strain selection [4,5]; (2) limited cross-reactivity against drifted IBV strains due to poor induction of broadly neutralizing antibodies (bnAbs) [6]; and (3) moderate efficacy (40–60%) in high-risk groups [7–9]. Current mRNA-based influenza vaccines require high doses (e.g. Moderna’s 50–100 μg quadrivalent vaccine) to achieve seroprotection, raising concerns about cost and reactogenicity [10,11].

Self-amplifying RNA (saRNA) vaccines, derived from alphavirus genomes, encode both the target antigen and viral RNA replication machinery, enabling intracellular amplification of RNA copies and prolonged antigen expression [12]. Clinical studies of SARS-CoV2 vaccines showed that a 5 μg dose of saRNA elicited humoral responses superior to those induced by a 30 μg dose of the conventional Comirnaty (BNT162b2) mRNA vaccine [13,14]. While clinical validation remains pending, circular RNA (circRNA) exhibits unique advantages as a next-generation RNA platform for influenza prophylaxis. Unlike linear mRNA requiring nucleoside modification to evade innate sensing, circRNA's covalently closed structure confers intrinsic resistance to exonuclease degradation, enabling prolonged antigen expression [15]. Recent studies indicate that circRNA vaccines encoding multivalent neuraminidase (NA) antigens provoke robust cross-reactive antibody responses and Th1-biased cellular immunity in mice, offering broad protection against both homologous and heterogeneous strains [16].

Our group previously established a nucleoside-modified mRNA vaccine platform that served as the technological foundation for COVID-19 vaccine development. The candidate (RQ3033, RNAcure) exhibited strong protective efficacy in Phase III clinical trials and subsequently received Emergency Use Authorization (EUA) in China in December 2023. This achievement validated the platform's capacity to rapidly generate protective immunity against emerging pathogens [17]. Here, we comprehensively evaluated mono and trivalent formulations using hemagglutinin (HA) antigens from the WHO-recommended 2023–2024 Northern Hemisphere strains (A/Wisconsin/67/2022 (H1N1, W67), A/Darwin/6/2021 (H3N2, D6), and B/Austria/1359417/2021 (Victoria, AUT21)). Our findings align with previous clinical results, showing suboptimal efficacy against the IBV. Then, we applied two next-generation RNA vaccine platforms, including saRNA and circRNA, for seasonal influenza vaccination, integrating subtype-specific sequence optimization strategies and systematically comparing immunogenicity and protective efficacy. We discovered that the saRNA platform was a superior and dose-reducing platform for IBV. These findings provide a framework for rationally selecting RNA vaccine platforms based on pathogen characteristics and desired immune outcomes, advancing the development of next-generation influenza vaccines.

## Result

### Sequence optimization and development of mRNA-based seasonal influenza vaccines

First of all, to investigate whether sequence optimization can enhance HA expression and overcome the suboptimal efficacy of IBV vaccines, we used the HA sequence from A/Puerto Rico/8/1934 (H1N1) (PR8) as a template to assess the impact of different sequence optimization strategies on mRNA-based protein expression. A total of 6 sequence design tools were used, focusing on codon optimization and RNA stability, with a systematic analysis of key parameters like the codon adaptation index (CAI) and minimal free energy (MFE). The results showed that the wild-type PR8-HA sequence (PR8-HA-WT) had a CAI of 0.7 and an MFE of −419 kcal/mol. After optimizing for maximum CAI, the CAI increased to 1.0, while the free energy dropped to −657.2 kcal/mol. In comparison, an optimization strategy focused on MFE and RNA secondary structure stability led to a CAI of 0.75 and further decreased the free energy to −1103.8 kcal/mol. Additional sequences with varying CAI and MFE parameters were also tested for expression analysis (Table S1). After transfecting BHK21 cells with mRNAs designed using various optimization methods, Western blot (WB) analysis revealed that both the CAI- and MFE-optimized PR8-HA sequences increased HA protein expression efficiency by approximately 40% (Figure S1A).

We then applied the sequence optimization strategies to design mRNAs for seasonal influenza viruses. mRNA sequences encoding HA genes from the WHO-recommended 2023–2024 influenza strains-A/Wisconsin/67/2022 (H1N1, W67), A/Darwin/6/2021 (H3N2, D6), and B/Austria/1359417/2021 (Victoria, AUT21)-were optimized using the maximal CAI and MFE strategies (Figure S1B). Agarose gel electrophoresis confirmed the uniformity of all three subtype-specific mRNA transcripts (Figure S1C, Table S2). Capillary electrophoresis further verified the integrity and expected size distribution of the optimized mRNA transcripts, showing a predominant full-length product with minimal detectable degradation or aberrant species across all three HA constructs (Figure S1D). Expression validation in BHK21 cells revealed that MFE-optimized mRNAs exhibited the highest protein expression for W67 and D6, whereas AUT21 showed comparable expression levels across optimization strategies (Figures S1E and S1F). Based on these results, we selected MFE-optimized constructs for subsequent vaccine production. Quantitative Western blot analysis, which calibrated with recombinant HA proteins (Standard curve, W67-HA: *Y* = 0.007625X–0.07734, *R*² = 0.9359; D6-HA: *Y* = 0.02268X + 0.09294, *R*² = 0.9795; AUT21-HA: *Y* = 0.006237X-0.01886, *R*² = 0.9951), confirmed efficient expression of all three HA subtypes by both mono- and trivalent (TRV) mRNA vaccines in BHK21 cells. Absolute quantification revealed higher expression levels of H3-D6-HA (0.47–1.69 ng/μL) and IBV-AUT21HA (0.26–0.60 ng/μL) compared to H1-W67-HA (0.21–0.41 ng/μL), a consistent observation across all transfection doses (250–1000 ng) (Figures S1H-J). These results demonstrate the effectiveness of sequence optimization and indicate sufficient IBV HA expression for vaccine development.

Next, the optimized mRNAs were encapsulated into SM102 lipid nanoparticles (LNPs), with subsequent physicochemical characterization demonstrating excellent formulation properties: all LNPs exhibited a particle size <100 nm, polydispersity index (PDI) < 0.1, and mRNA encapsulation efficiency >95% (Figure S1G), fully meeting the quality standards for vaccine delivery systems.

### Monovalent mRNA vaccines elicit dose-dependent humoral immunity in mice

To determine whether extensive sequence optimization could overcome the suboptimal efficacy against IBV on our mRNA platform, we systematically compared the immunogenicity of monovalent mRNA vaccines. BALB/c mice were immunized via a prime-boost regimen (3-week interval) with SM102 lipid-encapsulated mRNA-W67, mRNA-D6, or mRNA-AUT21 at three doses of 0.1, 1 μg, or 10 μg. An unrelated GFP mRNA-LNP served as a negative control, while a commercially available quadrivalent influenza vaccine (2 μg, split-virion, inactivated, QIV) was used as a positive control. Serum samples were collected at 3 weeks post prime and boost to evaluate antibody levels using enzyme-linked immunosorbent assay (ELISA) and microneutralization (MN) assays (Figure 1(A)). Both types of vaccines elicited high levels of IgG antibodies to vaccine-matched HA proteins after the prime immunization, with an enhancement following the boost (Figure 1(B–D)). A dose-dependent response was observed for all vaccines. Notably, mRNA-W67 and mRNA-D6 vaccines induced higher IgG levels than those of the QIV group, even at the low dose of 0.1 μg (Figure 1(B,C)). For IBV mRNA-AUT21, the 1 μg and 10 μg dose groups induced higher specific binding antibody levels compared to QIV (Figure 1(D)). To comprehensively assess vaccine-induced humoral immunity, we performed neutralization assays using (i) vaccine-matched strains (W67, D6, and AUT21) and (ii) antigenically distinct strains including A/Michigan/45/2015 (H1N1; MCG15; 98.06% HA gene homology with W67), A/Hong Kong/45/2019 (H3N2; HK19; 97.35% HA gene homology with D6), and B/Washington/02/2019 (Victoria lineage; WST19; 98.97% HA gene homology with AUT21) (Table S3). Neutralization assays demonstrated that the H1 and H3 (mRNA-W67 and mRNA-D6) vaccines elicited strong neutralizing antibody responses against their matched strains. At all tested doses, including 0.1 μg, neutralizing titres were significantly higher than those induced by QIV (Figure 1(E,F)). In contrast, no neutralizing antibodies against the matched IBV-AUT21 strain were detected at the 0.1 μg dose of the mRNA-AUT21 vaccine. However, higher doses (1 μg and 10 μg) elicited substantial titres that exceeded those of QIV (Figure 1(G)). All three mRNA vaccines at 1 μg and 10 μg doses elicited superior cross-neutralization against unmatched strains compared to QIV (Figure 1(H–J)). However, no detectable neutralizing antibodies against the B/Yamagata lineage strain were induced by the mRNA-AUT21 vaccine, suggesting limited cross-lineage neutralization despite robust activity against Victoria-lineage viruses (Figure S2A). In summary, all mRNAHA vaccines elicited strong, dose-dependent binding and neutralizing antibody responses. Notably, influenza A vaccines achieved high potency against matched strains even at low doses and demonstrated superior cross-neutralization breadth against unmatched strains at higher doses compared to the QIV.

**← Figure 1.**
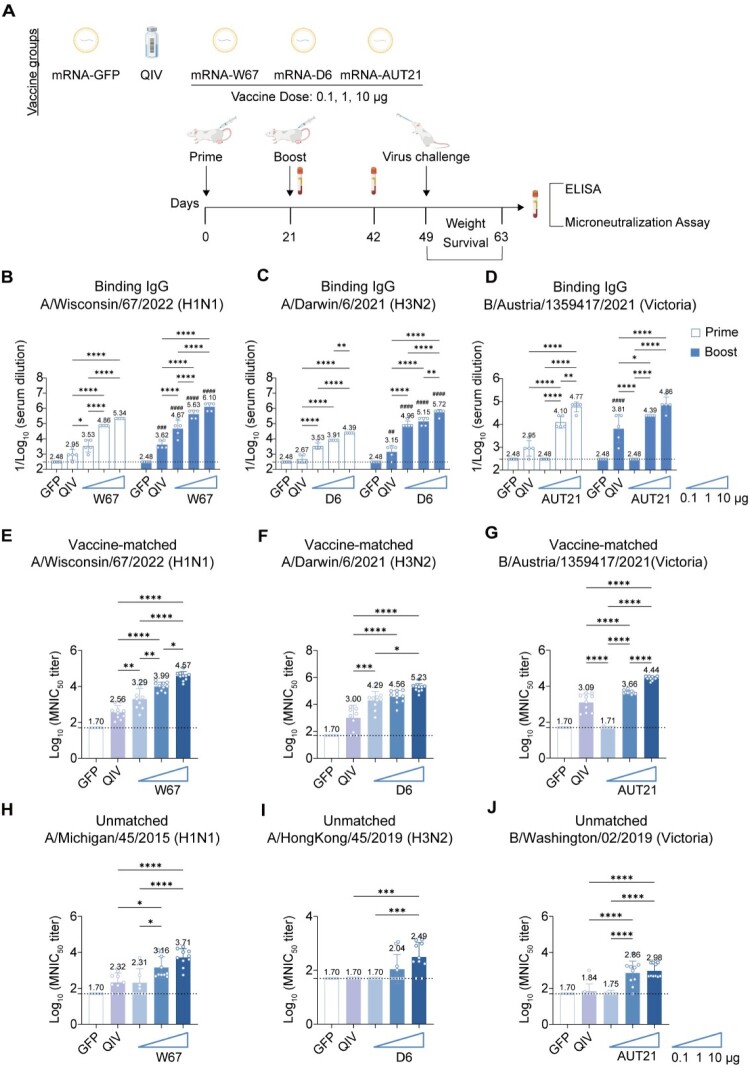
Humoral immune responses induced by monovalent mRNA vaccines. (A) Immunization scheme: Mice received prime-boost intramuscular immunizations (3-week interval) with 0.1, 1, or 10 μg of SM102-formulated mRNAs encoding W67 (H1N1), D6 (H3N2), or AUT21 (BV), 10 μg of GFP-encoding mRNA (control), or 2 μg of quadrivalent influenza vaccine (QIV, control). Sera were collected at week 3 and 6, with viral challenge performed at week 7. (B–D) HA-specific IgG ELISA (*n* = 5). Serum binding antibodies against recombinant (B) W67-H1, (C) D6-H3, and (D) AUT21-BV proteins were measured at 3 weeks post prime and boost immunization. (E–G) Homologous neutralization (*n* = 10). Post-boost (week 6) serum neutralizing titres (NT50) against (E) H1-W67, (F) H3-D6, and (G) BV-AUT21 viruses were measured using Microneutralization (MN) assays. (H–J) Heterologous neutralization (*n* = 10). Neutralizing activity against mismatched strains: (H) A/Michigan/45/2015 (H1N1) (MCG15), (I) A/HongKong/45/2019 (H3N2) (HK19), and (J) B/Washington/02/2019 (Victoria) (WST19) were measured. Data represent mean (SD); significance by two-tailed *t*-test (*p < 0.05, **p < 0.01, ***p < 0.001, ****p < 0.0001; prime *vs.* boost #*p* < 0.05, ##*p* < 0.01, ###*p* < 0.001, ####*p* < 0.0001).

**Figure 2.**
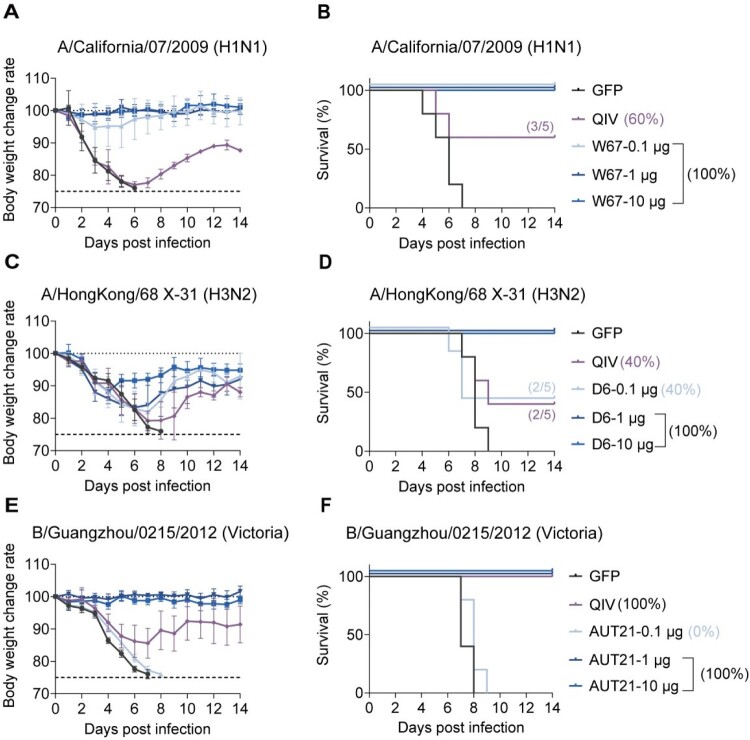
Protective of monovalent mRNA vaccines against influenza challenge. Mice were vaccinated (i.m.) with 0.1, 1, or 10 µg mRNA vaccine using a prime-boost schedule with an interval of 3 weeks. 4 weeks post boost, mice (*n* = 5) were challenged with 5×LD_50_ of H1-CA07, H3-HK68 or BV-GZ12. (A, C, E) Body weight changes. Data are presented as the mean (SD). (B, D, F) Survival curves. Data were analysed using a log rank test.

To further assess the immunogenic efficacy of the mRNA vaccines, we examined their protective effects against viral challenges using different influenza strains: (i) homologous strains, including A/California/07/2009 (H1N1; CA07; 96.82% HA gene homology with W67) and B/Guangzhou/0215/2012 (Victoria; GZ12; 98.46% HA gene homology with AUT21), and (ii) heterologous strains, including A/HongKong/68 (H3N2; HK68; 91.17% HA gene homology with D6), and B/Lee/1940 (B-Lee40; 95.89% HA gene homology with AUT21) (Table S3). The viral challenge studies showed that 0.1 μg of the H1 (mRNA-W67) vaccine provided 100% protection against CA07, with vaccinated mice maintaining stable body weight, compared to only 60% survival in the QIV group (Figure 2(A,B)). Both 1 μg and 10 μg doses of H3 (mRNA-D6) provided full protection against HK68, significantly outperforming QIV's 40% survival rate (Figure 2(C,D)). For IBV challenges, 1 μg and 10 μg of mRNA-AUT21 effectively protected against GZ12 (Figure 2(E,F)), with more stable body weight compared to QIV's transient weight loss. The 1 μg and 10 μg doses of mRNA-AUT21 achieved 20% and 60% protection against the heterologous Lee40 strain, while QIV offered no protection (Figures S2A and S2B). These findings demonstrate that influenza A vaccines achieve superior protective efficacy and enhanced dose efficiency compared to QIV.

WHO global influenza surveillance data (2022–2024) showed that H1N1 and BV strains maintained stable antigenic characteristics, while H3N2 exhibited significant antigenic drift, leading to the update of the H3N2 component in the 2024–2025 vaccine recommendations to A/Massachusetts/18/2022 (H3N2) (MC22). In a parallel evaluation, immunization with 10 μg mRNA-MC22 induced comparable HA-specific IgG levels to those achieved by mRNA-D6 (Figures S3A-D), demonstrating consistent immunogenicity across different H3N2 variants in our mRNA platform. Meanwhile, we also assessed the neutralizing antibodies in sera from mRNA-MC22 immunized mice against the HK14, HK19, and D6 strains (Figures S3E-G). We found that MC22 was also able to induce effective neutralizing antibodies against the D6 strain. Moreover, the mRNA-MC22 vaccine at 10 μg dose conferred complete protection against HK68 challenge (Figures S3H and S3I).

### Trivalent mRNA vaccine shows dose-dependent effects in mice, with suboptimal antibody response and protection against IBV at low doses

To assess the immunogenicity against IBV under multivalent vaccine conditions, we formulated a trivalent mRNA vaccine (mRNA-TRV) by combining monovalent mRNA-W67-LNP, mRNA-D6-LNP, and mRNA-AUT21-LNP at equal ratios. The immunization protocol was identical to that of monovalent vaccines, with doses of 0.1, 1, and 10 μg and a prime-boost regimen at 3-week intervals (Figure 3(A)). Serological evaluation demonstrated that mRNA-TRV induced dose-dependent increases in H1 HA-specific antibodies. The 0.1 μg low-dose group already showed significantly higher antibody titres compared to the QIV group, with even greater enhancement observed following booster immunization (Figure 3(B)). A similar dose-response pattern was observed for H3 HA antibodies, where all immunized groups displayed significantly elevated levels compared to QIV controls (Figure 3(C)). For IBVHA antibodies, significantly higher responses were only achieved in the 1 μg and 10 μg dose groups relative to QIV (Figure 3(D)).

**← Figure 3.**
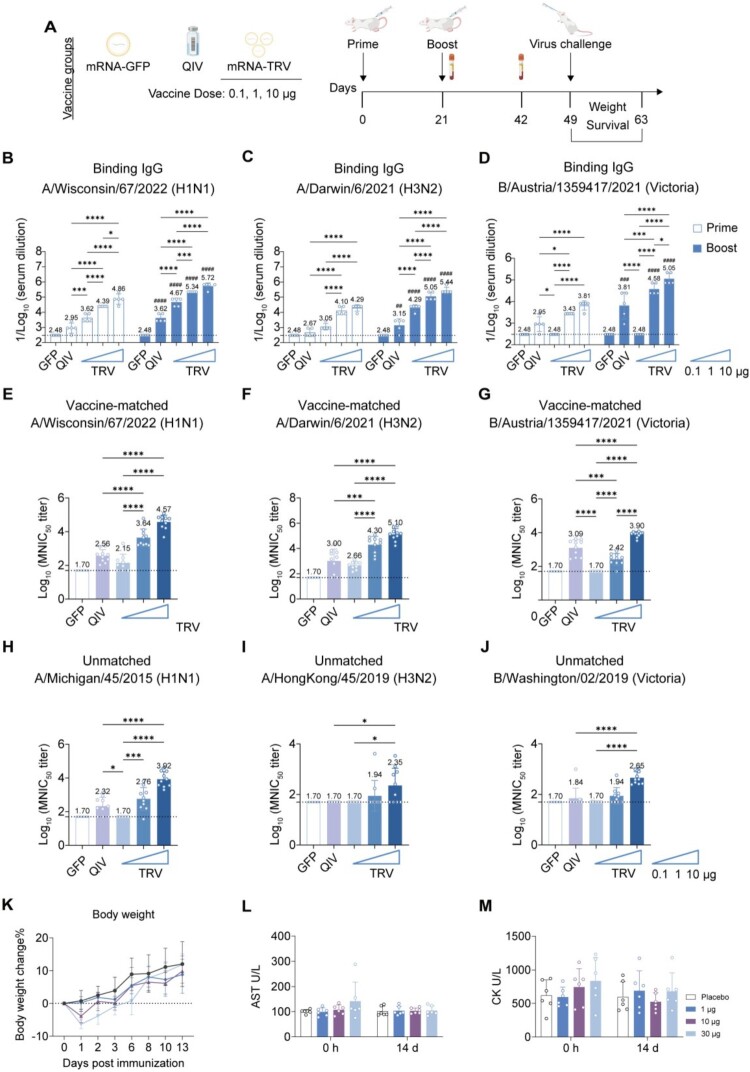
Humoral immune responses induced by trivalent mRNA vaccines. (A) Mice received prime-boost intramuscular immunizations (3-week interval) with 0.1, 1, or 10 μg of mRNA-TRV, 10 μg of GFP-encoding mRNA (control, same as Figure 2), or 2 μg of QIV (control, same as Figure 2). (B–D) HA-specific IgG ELISA (*n* = 5). Serum binding antibodies against recombinant (B) W67-H1, (C) D6 H3, and (D) AUT21-BV proteins were measured at week 3 and 6. (E–G) Homologous neutralization (*n* = 10). Post-boost (week 6) serum neutralizing titres (NT50) against (E) H1-W67, (F) H3-D6, and (G) BV-AUT21 viruses. (H–J) Heterologous neutralization (*n* = 10). Neutralizing activity against mismatched strains (H) H1MCG15, (I) H3-HK19, and (J) BV-WST19. (K) Body weight changes of mice after immunization. Body weight was monitored for 13 days after the first immunization to evaluate vaccine tolerability. (L–M) Serum biochemical safety assessment after immunization. Serum levels of (L) aspartate aminotransferase (AST) and (M) creatine kinase (CK) were measured at the indicated time points. Data represent mean (SD); significance by two-tailed *t*-test (*p < 0.05, **p < 0.01, ***p < 0.001, ****p < 0.0001; prime vs. boost #*p* < 0.05, ##*p* < 0.01, ###*p* < 0.001, ####*p* < 0.0001).

Neutralization assays demonstrated that the 1 μg and 10 μg dose groups of mRNA-TRV produced significantly higher neutralizing titres against matched strains (W67 and D6) for influenza A than the QIV group. Additionally, only the 10 μg dose group showed enhanced neutralizing titres against the IBV strain (AUT21) (Figure 3(E–G)). Crossneutralization evaluation against mismatched strains revealed a broad-spectrum neutralizing activity of the 10 μg mRNA-TRV dose against MCG15, HK19, and WST19, with particularly superior neutralization potency against the mismatched B strain compared to the QIV group (Figure 3(H–J)). However, no detectable neutralizing antibodies against the B/Yamagata strain were induced by mRNA-TRV (Figure S4A).

To further assess the safety profile of mRNA-TRV, an independent safety evaluation was performed using a simplified immunization schedule (Figure S5A). No obvious abnormal body weight changes were observed during the post-immunization monitoring period, indicating that mRNA-TRV was well tolerated across all tested doses (Figure 3(K)). Consistently, serum biochemical analysis showed no evident treatment-related changes in aspartate aminotransferase (AST) or creatine kinase (CK) levels after immunization (Figure 3(L,M)). Moreover, additional serum biochemistry parameters, including alanine aminotransferase (ALT), alkaline phosphatase (ALP), total protein (TP), albumin (ALB), creatinine (Crea), and lactate dehydrogenase (LDH), remained comparable between placebo and mRNA-TRV immunized groups at all tested doses (Figures S5B-G), further supporting the favourable safety profile of the trivalent mRNA vaccine.

**Figure 4.**
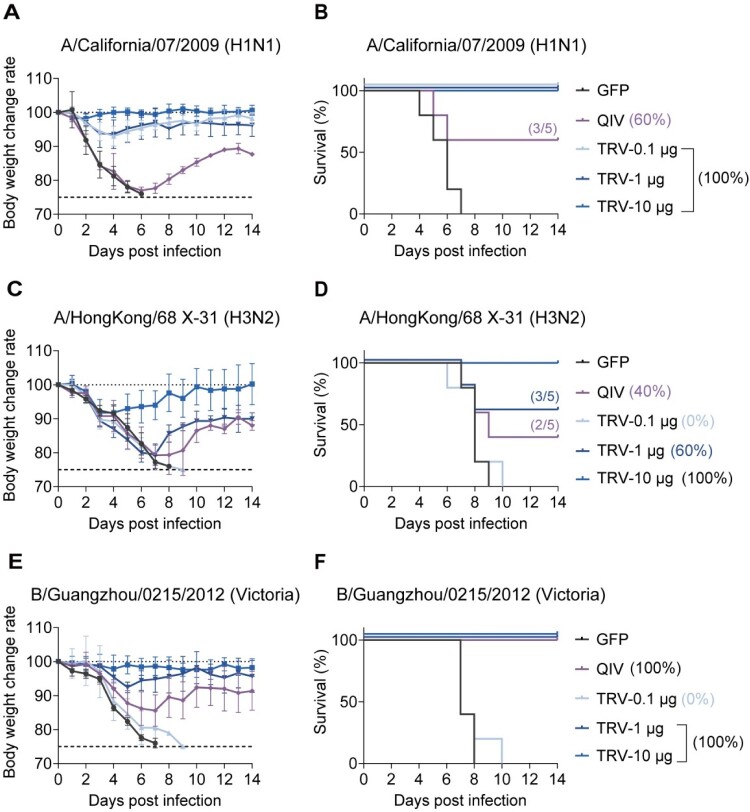
Protective of trivalent mRNA vaccines against influenza challenge. Mice were vaccinated (i.m.) with 0.1, 1 or 10 µg mRNA vaccine using in a prime-boost schedule with an interval of 3 weeks. 4 weeks post boost, mice (*n* = 5) were challenged with 5×LD_50_ of H1-CA07, H3-HK68 or BV-GZ12. (A, C, E) Body weight changes. Data are presented as the mean (SD). (B, D, F) Survival curves. Data were analysed using a log rank test.

Viral challenge experiments showed markedly better protection by mRNA-TRV against CA07 compared to QIV in all dose groups, including the 0.1 μg, which achieved 100% survival with preserved body weight. In contrast, the QIV group showed only 60% survival (Figure 4(A,B)). For HK68 challenge, complete protection was observed with 10 μg mRNA-TRV, while the 1 μg group showed 60% survival versus QIV's 40% (Figure 4(C,D)). In IBV challenges, both 1 μg and 10 μg doses of mRNA-TRV elicited complete protection against IBV (GZ12) in mice, accompanied by only moderate transient weight loss. Conversely, QIV-immunized mice, while achieving 100% survival, exhibited exacerbated weight loss (Figure 4(E,F)), underscoring the superior capacity of mRNA-TRV to mitigate clinical symptoms during IBV infection. Meanwhile only the 10 μg dose of mRNA-AUT21 induced 40% protection against the heterologous Lee40 strain (Figures S4A and S4B); all other groups failed to provide any detectable protection. The results indicate that mRNA-TRV confers complete protection against H1N1 influenza virus infection at doses as low as 0.1 μg, demonstrating markedly superior efficacy relative to the QIV. However, low-dose mRNA-TRV elicited only weak humoral immunity and offered limited protection against IBV, aligning with prior clinical observations [10].

### saRNA elicits superior immunity and confers complete protection against influenza B at a low dose

To address the challenge of high dosage requirements in conventional mRNA vaccines, particularly for IBV strains, we successfully synthesized seasonal influenza saRNA vaccines (Figure S6A) and circRNA vaccines (Figure S6E). These platforms are designed to overcome dosage limitations by leveraging their intrinsic capacity for low-dose efficacy and prolonged antigen expression. Subsequent Western blot analysis demonstrated relatively stable protein expression in cells over time from saRNA vaccines. Sequence optimization strategies were systematically evaluated for influenza HA antigens expressed via saRNA. Expression analysis identified CAI as the optimal method for W67 and AUT21 strains, while MFE optimization proved to be most effective for the D6 variant (Figures S6B and S6C). CircRNAs were co-circularized during *in vitro* transcription and purified via high-performance size-exclusion chromatography. E-gel EX electrophoresis systems (Thermo Fisher), which are able to distinguish circRNAs from other RNA species, showed relatively low circRNA formation rates and substantial premature termination products with the MFE optimization method (Figure S6F), suggesting its incompatibility with the circRNA platform. Western blot analysis subsequently revealed superior expression from CAI-optimized circRNA-W67 and circRNA-D6, while circRNA-AUT21 performed better with wild-type (WT) sequence (Figure S6G). Encapsulation of saRNA or circRNA using SM102 lipid yielded vaccines with favourable physicochemical properties: all formulations exhibited particle sizes <100 nm, PDI values <0.1, and encapsulation efficiencies >95%, confirming excellent nanoparticle uniformity (Figures S6D and S6H).

**Figure 5.**
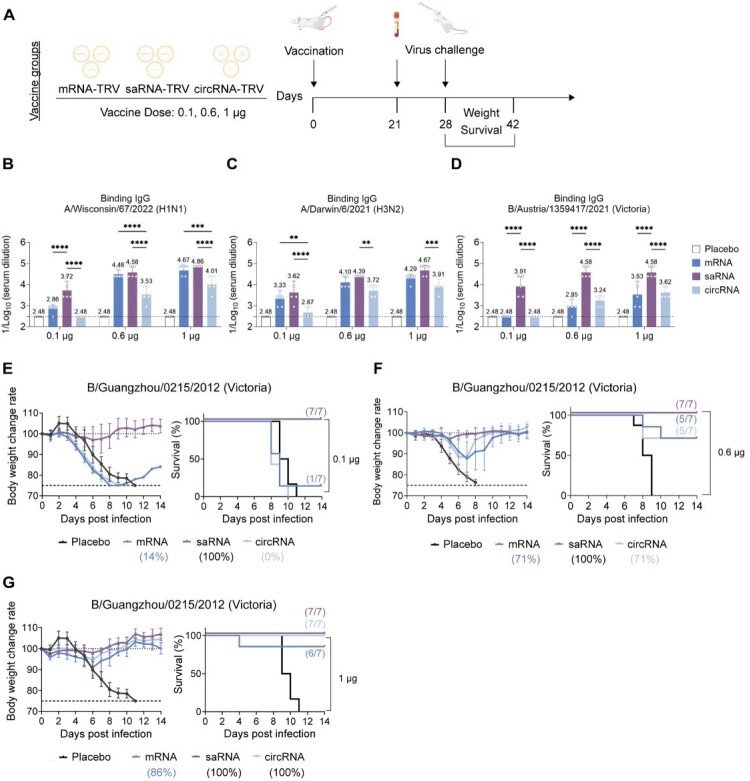
Humoral immune responses and protective efficacy induced by singledose trivalent RNA vaccines. (A) Immunization scheme. Mice received a single intramuscular (i.m.) immunization with 0.1, 0.6, or 1 µg of SM102-formulated mRNA-TRV, saRNA-TRV, circRNA-TRV, or a placebo control. Sera were collected at week 3 postimmunization, followed by viral challenge at week 4. (B–D) HA-specific IgG ELISA (*n* = 6). Serum binding antibodies against recombinant (E) W67-H1, (F) D6 H3, and (G) AUT21-BV proteins were measured at week 3. (E–F) Protective efficacy against viral challenge. Mice (*n* = 7) were immunized (i.m.) with 0.1, 0.6, or 1 µg of mRNA-TRV, saRNA-TRV, or circRNA-TRV in a single-dose regimen. At week 7, mice were challenged with 5×LD_50_ of BV-GZ12. Data represent mean (SD); significance by two-tailed *t*-test (*p < 0.05, **p < 0.01, ***p < 0.001, ****p < 0.0001).

To evaluate the efficacy of a single low-dose vaccination regimen reflecting real-world implementation, we prepared trivalent formulations by combining saRNA-W67-LNP, saRNA-D6-LNP, and saRNA-AUT21-LNP at a 1:1:1 ratio. Parallel test groups consisted of equivalent mixtures of mRNA and circRNA constructs for comparative analysis (Figures S7A and S7B). Following a single immunization at doses of 0.1 μg, 0.6 μg, or 1 μg in mice (Figure 5(A)), serological analysis of trivalent influenza vaccines encoding HA antigens revealed distinct dose-dependent and strain-specific immune profiles across mRNA, saRNA, and circRNA platforms (Figure 5(B–D)). At a dose of 0.1 μg, the antibody responses induced by saRNA for H1 and IBV strains were significantly higher than those induced by mRNA and circRNA. For H3, saRNA also demonstrated significantly higher antibody levels compared to circRNA, while remaining comparable to mRNA. At the intermediate dose of 0.6 μg, saRNA remained superior in inducing binding antibody responses, particularly against H3 and IBV antigens, whereas mRNA and circRNA showed more limited increases. At the 1 µg dose, the antibody levels induced by mRNA for H1 were comparable to those of saRNA. However, for H3 and IBV strains, saRNA induced significantly higher antibody levels compared to both mRNA and circRNA. In summary, a single low-dose immunization with saRNA-TRV at 0.1 µg was sufficient to induce robust humoral responses, particularly against the IBV.

Challenge studies showed 100% protection against homologous IBV strain (GZ12) in the 0.1 μg saRNA-TRV group with stable body weight, whereas all circRNA-TRV immunized mice succumbed by day 10 and mRNA-TRV achieved only 14% survival (Figure 5(E)). At the 0.6 μg dose, both saRNA-TRV and circRNA-TRV achieved complete protection, while mRNA-TRV provided partial protection with 71% survival (Figure 5(F)). At 1 μg, both saRNA-TRV and circRNA-TRV provided full protection, while mRNA-TRV showed 86% survival (Figure 5(G)).

**Figure 6.**
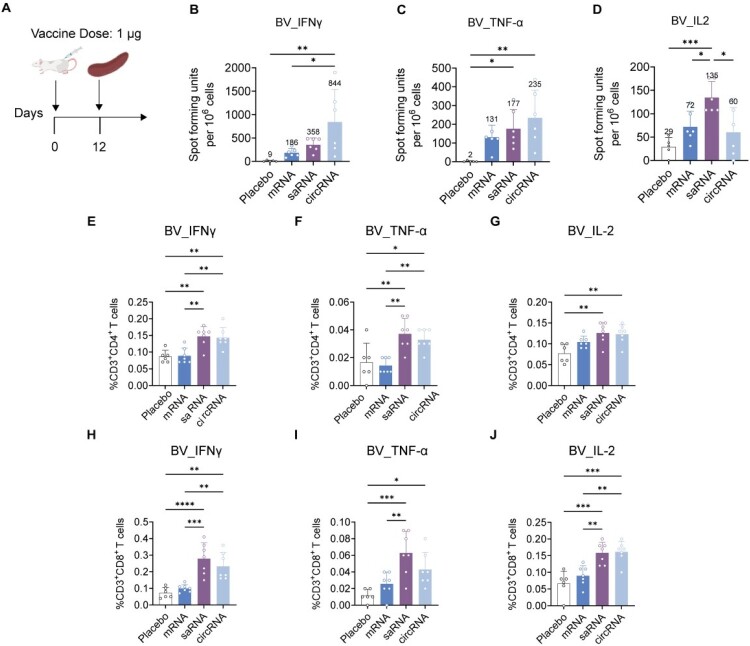
Cellular immune responses against BV-AUT21 induced by single-dose trivalent RNA vaccines. (A) Schematic illustration of the cellular immune evaluation protocol. Mice received a single intramuscular immunization with 1 μg of mRNA-TRV, saRNA-TRV, or circRNA-TRV, and splenocytes were collected on day 12 post-immunization for immunological analysis. (B–D) ELISpot analysis of antigen-specific T cell responses following stimulation with BV-AUT21 peptide pool. Numbers of cytokine-secreting cells producing (B) IFN-γ, (C) TNF-α, and (D) IL-2 are shown. (E-G) Flow cytometric analysis of BV-AUT21-specific CD4+ T cell responses. Frequencies of cytokine-positive CD3 + CD4+ T cells producing (E) IFN-γ, (F) TNF-α, and (G) IL-2 are shown. (H–J) Flow cytometric analysis of BV-AUT21-specific CD8+ T cell responses. Frequencies of cytokine-positive CD3 + CD8+ T cells producing (H) IFN-γ, (I) TNF-α, and (J) IL-2 are shown. Data represent mean (SD); significance by twotailed *t*-test (*p < 0.05, **p < 0.01, ***p < 0.001, ****p < 0.0001).

We further conducted a systematic assessment of the cellular immune responses induced by single-dose immunization. On day 12 post-immunization, splenocytes were harvested from mice that had received a 1 μg dose of mRNA-TRV, saRNA-TRV, or circRNA-TRV according to the immunization scheme shown in Figure 6(A). Following stimulation with peptide pools H1-W67, H3-D6, and BV-AUT21, IFN-γ, TNF-α, and IL-2 secretion was quantified by ELISpot (Figures S8A-C, J-L, and Figure 6(B–D)), and antigen-specific CD4+ and CD8+ T cell responses were further evaluated by intracellular cytokine staining and flow cytometry (Figures S8D-I, S8M-R, and Figure 6(E–J)). All three platforms elicited antigen-specific T cell responses; however, the response magnitude and cytokine profiles exhibited pronounced platform dependence and antigen subtype specificity. Specifically, under H1-W67 stimulation, mRNA-TRV and saRNA-TRV induced significantly higher IFN-γ levels than circRNA-TRV, and only these two platforms triggered a significant increase in IL-2 (Figures S8A-C). Flow cytometric analysis further showed that H1-specific cytokine-producing CD4+ and CD8+ T cell responses were generally more pronounced in the mRNA-TRV and saRNA-TRV groups than in the circRNA-TRV group (Figures S8D-I). For the H3-D6 antigen, saRNA-TRV elicited the strongest IFN-γ response, whereas circRNA-TRV did not induce detectable TNF-α release (Figures S8J-L). Consistently, intracellular cytokine staining showed enhanced H3-specific CD4+ and CD8+ T cell activation in the saRNA-TRV group, particularly for IL-2 and TNF-α production (Figures S8M-R). Notably, under BV-AUT21 stimulation, circRNA-TRV yielded the highest IFN-γ levels, while saRNA-TRV selectively induced the strongest IL-2 response, significantly surpassing both mRNA-TRV and circRNA-TRV (Figure 6(B–D)). Flow cytometry further demonstrated that BV-AUT21 stimulation induced measurable cytokineproducing CD4+ and CD8+ T cell populations in all three vaccine groups, with platform-dependent differences in cytokine preference and response magnitude (Figure 6(E–J)). Taken together, saRNA demonstrated superior humoral immunogenicity to mRNA and circRNA under low-dose immunization conditions and conferred robust protective efficacy against homologous IBV challenge, highlighting its potential as an effective platform for inducing protective immunity at reduced doses, particularly against IBV.

### Enhanced immunity by saRNA-TRV in prime-boost vaccination

**Figure 7.**
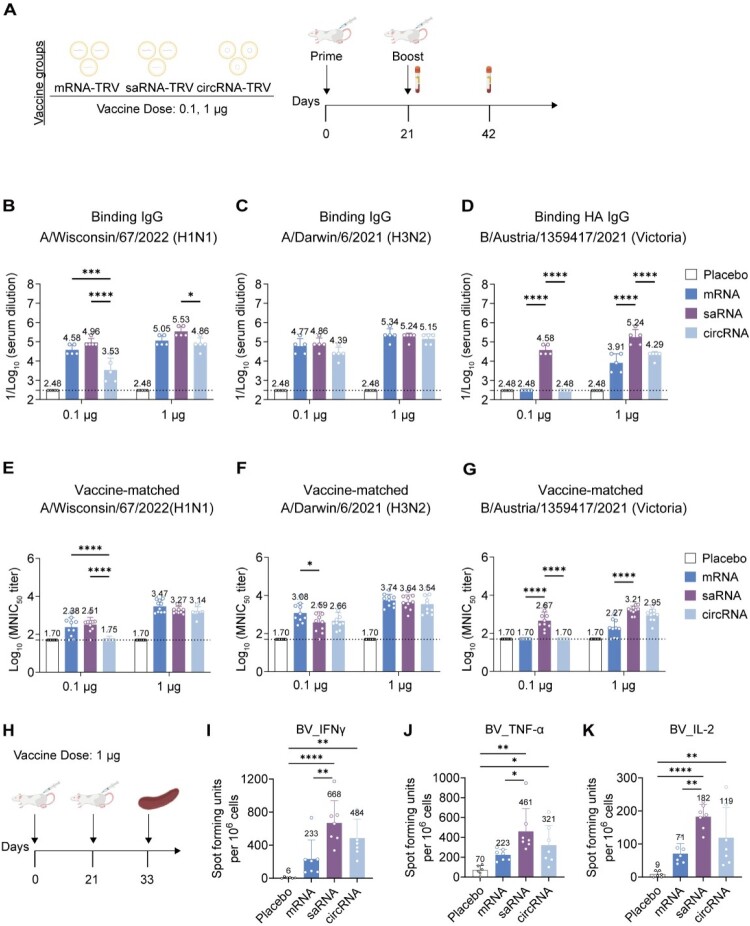
Evaluation of prime-boost RNA vaccine immunization across different platforms. (A) Immunization scheme. Mice received prime-boost intramuscular immunization with 0.1 μg or 1 μg of SM102-formulated mRNA-TRV, saRNA-TRV, or circRNA-TRV or a placebo control. Sera were collected at weeks 3 and 6. (B–D) HA-specific IgG ELISA (*n* = 5). Serum binding antibodies against recombinant (B) W67 H1, (C) D6-H3, and (D) AUT21-BV proteins were measured at week 3 or 6. (E–G) Homologous neutralization (*n* = 10). Post-boost (week 6) serum neutralizing titres (NT50) against (E) H1-W67, (F) H3-D6, and (G) BV-AUT21 viruses. (H) Immunization scheme: Mice were immunized intramuscularly with 1 μg of mRNATRV, saRNA-TRV, or circRNA-TRV with a 3-week interval between doses. Spleens were collected and lymphocyte suspensions were prepared on day 12 post-boost. (IK) Number of T cells secreting with BV-AUT21 peptide pool. Data represent mean (SD); significance by two-tailed *t*-test (*p < 0.05, **p < 0.01, ***p < 0.001, ****p < 0.0001).

We further investigated the immune responses of a prime-boost vaccination strategy. As outlined in Figure 7(A), mice received a booster shot at 3 weeks post-prime. Serological analysis revealed significant antibody response enhancement following booster immunization. At the 0.1 μg dose, saRNA-TRV induced significantly higher H1 HA-binding antibody titres compared to mRNA-TRV and circRNA-TRV. At the 1 μg dose, all three platforms elicited comparable levels of H1 HA-binding antibodies (Figure 7(B)). Regarding the matched H3 HA antigen, saRNA-TRV demonstrated similar humoral immune responses at both 0.1 μg and 1 μg doses across all platforms (Figure 7(C)). Notably, saRNA-TRV showed superior immunogenicity in response to the IBV antigen at both doses, underscoring its potential for the development of IBV vaccines (Figure 7(D)).

To further evaluate the functional relevance of binding antibody responses, we assessed neutralizing antibody titres against matched viral strains. Consistent with the binding antibody data, saRNA-TRV at a 0.1 μg dose produced neutralization titres similar to those of mRNA-TRV against the matched strains (W67), with no significant differences observed at the 1 μg dose (Figure 7(E)). For the H3 matched strains (D6), neutralization titres across platforms showed only marginal differences (Figure 7(F)). Notably, saRNA-TRV maintained its immunogenicity advantage against IBV (AUT21), with neutralization titres surpassing those of mRNA at both dose levels (Figure 7(G)).

We next implemented a booster immunization strategy, and splenocytes were collected on day 12 post-boost for the same stimulation and downstream analyses (Figure 7(H)). Under H1-W67 stimulation, all platforms robustly produced IFN-γ, TNF-α, and IL-2, with no significant differences among groups (Figures S9A-C). Under H3-D6 stimulation, the induction of IFN-γ by mRNA-TRV was superior to that of saRNA-TRV and circRNA-TRV (Figures S9D-F). Notably, under BV-AUT21 stimulation, saRNA-TRV induced significantly higher ELISpot responses than mRNA-TRV across all three cytokines (Figure 7(I–K)).

These results collectively demonstrate that saRNA-TRV, particularly at lower doses, induces superior neutralizing antibody responses and T cell immunity, further supporting its potential as a versatile platform for IBV vaccine development.

### saRNA vaccines elicit durable binding antibody responses

**Figure 8.**
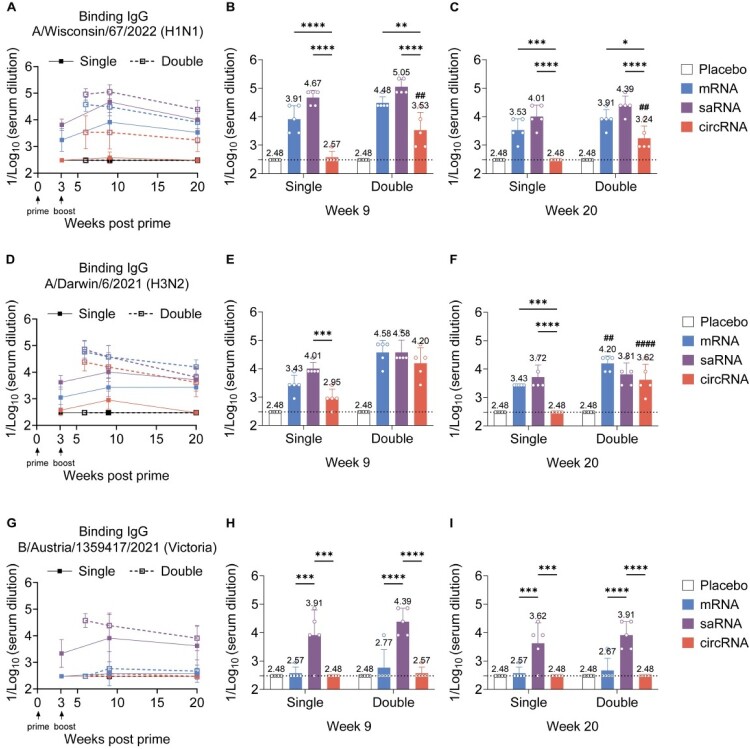
Detection of long-term antibodies following a 0.1 μg dose of mRNA, saRNA, and circRNA vaccines. Serum samples were collected from mice at weeks 3, 6, 9 and 20 after the initial immunization. A comparison was made between single-dose and double-dose immunization strategies, with administered doses of HA-specific IgG ELISA (*n* = 5). Serum binding antibodies against recombinant (A–C) W67-H1, (D-F) D6-H3, and (GI) AUT21-BV proteins were measured. Data represent mean (SD); significance by two-tailed *t*-test (*p < 0.05, **p < 0.01, ***p < 0.001, ****p < 0.0001; single vs. double #*p* < 0.05, ##*p* < 0.01, ###*p* < 0.001, ####*p* < 0.0001).

Previous studies suggest that saRNA can sustain effective antibody production *in vivo* [18]. To further verify whether this RNA platform exhibits such immunological characteristics, we employed single-dose and prime-boost strategies, collected mouse sera at weeks 3, 6, 9, and 20 after the initial immunization, and measured binding antibodies to the W67 antigen by ELISA. At the 0.1 μg dose, regardless of single-dose or prime-boost immunization, mRNA and saRNA vaccines induced significantly higher antibody levels at weeks 9 and 20 than the circRNA vaccine. Moreover, under the prime-boost regimen, the circRNA-TRV group showed significantly higher antibody levels than its single-dose group, suggesting that at 0.1 μg, a single dose of circRNA may be insufficient to elicit a strong antibody response (Figure 8(A–C)). At the 1 μg dose, antibody levels in the single-dose groups rose from week 3 to week 9 but declined by week 20, with no significant differences among the three RNA vaccines; in the prime-boost groups, all three vaccines showed a decline in antibody levels by week 20 (Figures S10A-C).

The binding antibody responses to the D6 antigen largely mirrored those observed for W67. At both 0.1 μg and 1 μg, single-dose immunization with mRNA, saRNA, and circRNA led to increased antibody levels by week 9 followed by a gradual decline by week 20, whereas under prime-boost conditions, antibody levels declined continuously from week 3 onward (Figure 8(D–F)). Additionally, at 0.1 μg, single-dose saRNA induced significantly higher antibody levels at weeks 9 and 20 than circRNA. At 1 μg, there were no statistical differences among the three RNA vaccines under either singledose or prime–boost regimens; however, in the prime–boost condition, the saRNA-TRV group exhibited significantly higher antibody levels than its single-dose counterpart (Figures S10D-F).

For the influenza BV-AUT21-HA antigen, the pattern differed markedly from W67 and D6. At 0.1 μg, neither mRNA-TRV nor circRNA-TRV induced detectable antibody responses, whereas saRNA-TRV elicited significantly higher antibody levels than both mRNA-TRV and circRNA-TRV under single-dose and prime–boost conditions (Figure 8(G–I)). At 1 μg, there were no significant differences among the three RNA vaccines in the single-dose groups; however, in the prime–boost regimen, saRNA-TRV induced significantly higher antibody levels than mRNA-TRV at week 9, and by week 20, both saRNA-TRV and circRNA-TRV were significantly higher than mRNA-TRV (Figures S10G-I).

In summary, at a low dose (0.1 μg), the saRNA platform consistently induces the most durable and highest antibody responses across most antigens—particularly the conserved influenza HA antigen—highlighting the unique advantage of its self-amplifying mechanism in sustaining long-term immunity.

## Discussion

Although conventional inactivated seasonal influenza vaccines can elicit protective immune responses, their overall protective efficacy remains suboptimal [19–21]. In recent years, with groundbreaking advancements in mRNA technology, mRNA-based vaccines have emerged as a highly promising prophylactic approach [22–27]. However, current studies indicate that mRNA vaccines often require relatively high doses to achieve effective immune protection [28,29]. Our study systematically evaluated seasonal influenza vaccines based on mRNA, saRNA, and circRNA platforms, revealing distinct immunological characteristics and protective efficacy profiles among different nucleic acid vaccine platforms. The findings provide critical insights into sequence optimization strategies and platform-specific immune responses, particularly highlighting the exceptional protection of saRNA vaccines against IBV at ultralow doses.

The influenza mRNA vaccines in clinical trials showed lower efficacy against IBV, and a feasible solution to improve this is optimizing the mRNA sequence for higher protein expression levels. We comprehensively assessed CAI and MFE optimization strategies across platforms. For mRNA vaccines, MFE optimization significantly enhanced protein expression of H1N1 and H3N2 subtypes, while AUT21 showed less dependence on specific optimization approaches (Figure S1F), likely reflecting inherent structural differences among influenza subtypes. Notably, optimization strategies required platform-specific adaptation: CAI optimization favoured saRNA stability, whereas MFE optimization impaired circRNA circularization efficiency due to excessive secondary structure formation.

In addition to the immunogenicity data of seasonal influenza HA antigen obtained from the conventional mRNA vaccine platform, our study further evaluated the protective immune efficacy of the saRNA vaccine platform against IBV at ultra-low doses. The research yielded significant findings: the saRNA vaccine provided full protection against IBV/GZ12 strain challenge at an ultra-low dose of 0.1 μg. In contrast, the mRNA vaccine group showed much lower efficacy, with only one out of seven mice surviving marginally at the same dose. These results suggest that the saRNA vaccine may be more effective at such low doses, making it a promising candidate for further development (Figure 5). This remarkable dose-efficacy difference can be primarily attributed to the following mechanisms: Firstly, the unique self-replicating characteristic of saRNA enables sustained antigen expression in hosts. This prolonged antigen exposure not only significantly enhances HA-specific antibody production but also promotes antibody affinity maturation. Secondly, the double-stranded RNA intermediates generated during saRNA replication serve as natural adjuvants. By activating pattern recognition receptor pathways such as RIG-I/MDA5, they strongly induce type I interferons and pro-inflammatory cytokine secretion, thereby significantly enhancing adaptive immune responses (Figure S11) [30]. Furthermore, saRNA vaccination at ultra-low doses elicited robust antigen-specific cellular immunity, as evidenced by significantly higher IFN-γ and IL-2 production in splenocytes upon HA peptide stimulation compared with mRNA and circRNA vaccines. Such T cell responses, particularly the elevated IL-2 levels detected in BV-AUT21 stimulation, suggest that saRNA induces a more effective helper T-cell profile, supporting sustained B-cell activation and antibody generation. This synergy between humoral and cellular responses likely underlies the full protective effect observed in the challenge model.

However, despite the clear immunological and dose-sparing advantages observed for saRNA vaccines, several practical challenges should be considered for their large-scale translation. Compared with conventional mRNA, saRNA molecules are substantially longer and structurally more complex, which may reduce in vitro transcription efficiency and increase susceptibility to premature termination or the generation of heterogeneous by-products during manufacturing [31,32]. In addition, the self-replicating nature of saRNA, while beneficial for prolonged antigen expression *in vivo*, imposes stricter requirements on RNA integrity, sequence design, and formulation consistency, all of which may complicate process development and quality control at industrial scale [33]. These factors could create production bottlenecks and partially offset the economic advantages expected from dose sparing. Moreover, although the lower effective dose of saRNA has the potential to reduce cost per vaccination, this benefit must be balanced against potentially higher manufacturing complexity, more demanding purification procedures, and the need for robust control of innate immune activation and product stability [34]. Therefore, the real-world cost-effectiveness of saRNA vaccines will depend not only on their biological potency, but also on future advances in scalable production processes, analytical characterization, and formulation technologies.

These findings advance RNA vaccinology in multiple aspects: first, by proposing platform-specific optimization principles for RNA vaccine development; second, by demonstrating that saRNA vaccines at ultralow doses, particularly against IBV, can achieve protection comparable to or better than that of conventional mRNA vaccines, highlighting their substantial dose-sparing potential; and third, by providing a framework for rational platform selection based on pathogen characteristics, desired immune outcomes, and translational feasibility. Although further work is needed to address manufacturing scalability and cost-related challenges, the superior saRNA performance against IBV suggests considerable promise for future vaccine development. This advantage may reflect unique features of IBV biology, and further investigation may also inform vaccine design for other pathogens.

## Methods

### Bacterial strains and cell lines

*E. coli* DH5α cells (AlpalifeBio, Guangzhou, China) were used for plasmid cloning and cultured in LB broth (Sango) supplemented with or without ampicillin (100 μg/mL) or kanamycin (50 μg/mL). BHK-21, HEK293 T, MDCK and MDCK-STAT1KO cells were purchased from ATCC and cultured in Dulbecco’s modified Eagle’s medium (DMEM; BasalMedia, Gibco) supplemented with 10% heat-inactivated fetal bovine serum (FBS; Sigma-Aldrich) and 10 U/mL penicillin–streptomycin (Gibco) at 37 ℃ and 5% CO_2_.

### Viruses

The A/Hong Kong/68 (X-31) (H3N2) and B/Lee/1940 strains were propagated in 9- to 10-day-old specific pathogen-free (SPF) embryonated chicken eggs (Wens). Briefly, eggs were candled to confirm embryo viability before inoculation. Virus stock (1,000 FFU/egg) was injected into the allantoic cavity using a sterile needle. Inoculated eggs were incubated at 37℃ for 48–72 h, then chilled at 4℃ for ≥4 h to facilitate harvesting. The allantoic fluid was collected, clarified by centrifugation (3,000 ×g, 10 min, 4℃), aliquoted, and stored at −80℃.

The following strains were propagated in MDCK-STAT1 KO cells to enhance viral yield: A/Wisconsin/67/2022 (H1N2), A/Michigan/45/2015 (H1N1), A/Puerto Rico/8/1934 (H1N1), A/California/07/2009 (H1N1pdm09), A/Darwin/6/2021 (H3N2), A/Hong Kong/45/2019 (H3N2), B/Austria/1359417/2021(Victoria), B/Washington/02/2019 (Victoria), B/Guangzhou/0215/2012 (Victoria). Cells were infected at an MOI of 0.1 and incubated at 37℃, 5% CO_2_ for 48–72 h. Viral supernatants were harvested, centrifuged (3,000 g, 10 min, 4℃), aliquoted, and stored at −80℃. Viral titres were measured using a Fluorescent Focus Assay with MDCK cells (as detailed in the Fluorescent Focus Assay section). All viruses were sequenced to confirm that the correct sequences were retained during propagation.

### Fluorescent focus assay (FFA)

MDCK cells were seeded in 96-well plates at a density of 2.5 × 10⁴ cells/well using DMEM supplemented with 5% fetal bovine serum, and incubated overnight at 37℃ with 5% CO_2_ until reaching approximately 90% confluence. Virus stocks were serially diluted 10-fold in infection medium (DMEM containing 2 μg/mL TPCK-trypsin (Sigma)). Cell monolayers were washed once with PBS prior to inoculation with 100 μL of diluted virus per well. Following 1 h incubation at 37℃ with gentle rocking every 15 min to ensure uniform viral adsorption, the inoculum was removed and replaced with 100 μL of 1% CMC in MEM mixture. Plates were then incubated for 2842 h at 37℃ with 5% CO_2_ to allow viral replication. After incubation, cells were fixed with 4% paraformaldehyde in PBS for 1 h at room temperature, followed by permeabilization with 0.1% Triton X-100 in PBS for 30 min. After three washes with PBS, cells were blocked with 1% BSA in PBS for 1 h at room temperature. Immunostaining was performed using mouse anti-NP monoclonal antibody (4R5) (1:4000 dilution in 1% BSA/PBS) for 1 h, followed by three washes with PBST (PBS containing 0.05% Tween-20). HRP-conjugated goat anti-mouse IgG (1:2000 dilution in 1% BSA/PBS) was then applied and incubated for 1 h protected from light. Following three final washes with PBST, TMB One Solution(HRP-based WB) (YEASEN) was added for 10 min, and quantification was performed using an ELISpot reader (AT-Stop1100/CTL).

### RNA vaccine design and synthesis

The HA genes were derived from the WHO-recommended 2023–2024 Northern Hemisphere vaccine strains: H1N1 A/Wisconsin/67/2022 (GISAID: EPI_ISL_15928563), H3N2 A/Darwin/6/2021 (GISAID: EPI_ISL_3534319), and B/Victoria lineage B/Austria/1359417/2021 (GISAID: EPI_ISL_983345). Target sequences were optimized using mammalian codon bias adaptation and RNA secondary structure minimization algorithms to enhance translational efficiency and stability in mammalian cells. At the same time, we sought to improve mRNA secondary structure stability by maximizing the minimum free energy (MFE) while maintaining the original amino acid sequence. This was achieved by exploring synonymous substitutions within the coding region, leveraging the degeneracy of the genetic code. We employed the CDSfold algorithm to guide the identification of synonymous sequence. The software is available at https://github.com/gterai/CDSfold [35]. The mRNA was synthesized via *in vitro* transcription (IVT) from linearized DNA templates. These templates, which contained the human β-globin UTRs and a 100-nt poly(A) tail, were generated by PCR and purified (Promega gel extraction kit). The IVT setup included T7 RNA polymerase (APExBIO), pseudouridine (Ψ; APExBIO) in place of UTP to reduce immunogenicity, and a Cap1 analog (APExBIO) for 5’ capping. Post-transcription, the mixture was treated with DNase I (APExBIO) to remove the template DNA, followed by mRNA purification using oligo-dT chromatography [36]. The saRNA constructs contain the Venezuelan Equine Encephalitis Virus strain TC-83 5’ UTR, 3’ UTR, non-structural protein genes, and the influenza virus HA gene. The saRNA was synthesized by IVT as previously described for mRNA. circRNA was synthesized using the permuted intronexon (PIE) splicing strategy. The circularization protocol was performed as follows:

First, IVT was conducted at 47℃ for 2 h using thermostable T7 polymerase (APExBIO) to generate pre-circRNA. Following initial purification using conventional methods (which proved insufficient for removing precursor and nicked byproducts), the circularized products underwent additional purification through HPLC (Sepax) with elution in 1×TE buffer, followed by a final purification step using an RNA clean-up kit (Magen Biotechnology Co., Ltd., Guangzhou, China). The integrity and purity of mRNA and saRNA were assessed by TBE-urea gel electrophoresis, and mRNA was further characterized by capillary electrophoresis using the Agilent DNF-471 RNA Kit. The circularization efficiency of circRNA was assessed using the E-Gel system (Thermo Fisher).

### *In vitro* expression analysis

To assess protein expression, BHK-21 and HEK293T cells were cultured in 12-well plates until reaching 50–60% confluency. These cultures were then transfected with varying amounts (250, 500, or 1000 ng) of monovalent (MRV) or trivalent (TRV) mRNA vaccines, complexed with Lipo8000 transfection reagent (Beyotime) in OptiMEM reduced serum medium (Gibco). At 24 h after transfection, the cells were lysed using RIPA buffer supplemented with a protease inhibitor cocktail. The protein concentration in the resulting lysates was determined by a bicinchoninic acid (BCA) assay (Thermo Scientific). To enable precise quantification of HA expression, standard curves were generated using known concentrations of recombinant HA proteins (W67HA, D6-HA, and AUT21-HA; South China Vaccines). All cell lysate samples were subsequently normalized to specific loading concentrations based on these standardsW67 (20 µg/well), D6 (10 µg/well), and AUT21 (20 µg/well)-before proceeding to immunoblotting. The normalized protein samples were electrophoresed on 4–20% polyacrylamide gradient gels (ACE Biotechnology) and subsequently electrotransferred onto PVDF membranes (Millipore). After blocking with a 5% solution of non-fat dry milk, the membranes were incubated with specific primary antibodies targeting H1, H3, or BV HA proteins (all from Sino Biological) for one hour at ambient temperature. Following extensive washes, the blots were exposed to a horseradish peroxidase (HRP)-conjugated goat anti-human secondary antibody (Sangon Biotech; 1:5000) for another hour. Immunoreactive bands were detected by applying an enhanced chemiluminescence substrate (CoWin Biotech), and digital images were captured using an iBright CL750 system (Thermo Fisher). The band intensities were finally quantified through greyscale analysis with ImageJ software [36].

### LNP formulation and characterization

Lipid nanoparticle (LNP) formulation was prepared as follows: SM102 (Shenzhou Pharmaceutical), DSPC (Nippon Fine Chemical), cholesterol (Nippon Fine Chemical), and PEG2000-DMG (JenKem Technology) in absolute ethanol at a molar ratio of 50:10:38.5:1.5. This lipid mixture was then combined with RNA dissolved in 25 mM sodium acetate buffer (pH 5.0) using a Nano Precision microfluidic system (Shanghai RNACure Biopharma) with controlled flow parameters to ensure consistent nanoparticle formation. Immediately following mixing, the formulations were subjected to a two-fold dilution with the same sodium acetate buffer to optimize particle stability, then dialysed overnight at 4℃ against 20 mM Tris-acetate buffer (pH 7.5) using Slide-A-Lyzer dialysis cassettes with a 10 kDa molecular weight cutoff. After dialysis, the formulations were processed through sequential steps including concentration using Amicon Ultra centrifugal filters (Millipore), sterile filtration through 0.22 µm PVDF membranes (Merck), and comprehensive characterization. The nanoparticle size distribution and polydispersity index were determined by dynamic light scattering, while surface charge was measured via phase analysis light scattering using a Zetasizer Nano ZA system (Malvern Instruments). RNA encapsulation efficiency was quantitatively assessed using the Quant-iT RiboGreen assay (Thermo Fisher Scientific), with measurements taken both before and after Triton X-100 treatment to accurately determine the percentage of encapsulated RNA.

### Animal experiments

The vaccination procedures were conducted in strict accordance with the approved animal protocol (GZLAB-AUCP-2023-10-A7) from the Animal Care and Use Committee of Guangzhou Laboratory, China. The rodent housing facility maintained an ambient temperature of 22℃ ± 3℃ with relative humidity controlled between 30% and 70%. An automated 12-hour light/12-hour dark cycle was implemented throughout the study period.

### Immunization and challenge studies

For Figure 2, female BALB/c mice (7-week-old) were randomly allocated into experimental groups and immunized intramuscularly in the quadriceps with mRNALNP formulations at graded doses (0.1, 1, or 10 μg). Age-matched control mice received either 2 μg of 2023–2024 seasonal QIV. All immunizations followed a primeboost schedule with identical doses administered at 3-week intervals. Serial blood collections were performed via retro-orbital bleeding at 3 weeks post-prime and 3 weeks post-boost (week 6) for humoral immunity evaluation. Four weeks after the boost immunization, mice were challenged with a 5×LD_50_ dose of virus.

For the safety evaluation shown in Figure 3(K–M) and Figure S5, 7-week-old female BALB/c mice were intramuscularly immunized with a single dose of mRNA-TRV (1 μg, 10 μg, or 30 μg), with placebo-treated mice as controls. Body weight was monitored for 14 days after immunization, and blood samples were collected at 0 h and 14 d postimmunization for serum biochemical analysis, including AST, CK, ALT, ALP, TP, ALB, CREA, and LDH.

For Figure 6: Seven-week-old female BALB/c mice were intramuscularly immunized with either saRNA-LNP or circRNA-LNP (0.1 μg, 0.6 μg, or 1 μg), alongside control groups receiving mRNA-LNP at matched doses (0.1 μg, 0.6 μg, or 1 μg). Blood samples were collected at week 3 for immunological assessment. Four weeks after a single immunization, mice were challenged with a 5×LD_50_ dose of virus. Following infection, body weight was monitored daily for 14 days. Mice reaching the predefined humane endpoint (≥25% weight loss from baseline) were euthanized.

### Enzyme linked immunosorbent assay (ELISA)

Serum levels of antigen-specific IgG antibodies were measured using a standardized ELISA. Briefly, 96-well microplates were coated with 100 μL/well of recombinant HA protein (0.5 μg/mL in coating buffer) and incubated overnight at 4℃. After three washes with PBS, nonspecific binding sites were blocked with 200 μL/well of 5% nonfat milk in PBS for 1 h at 37℃. Serum samples and monoclonal antibodies were subjected to three-fold serial dilutions (initial dilution 1:300) in PBS. Following incubation and three additional washes, 100 μL/well of horseradish peroxidase (HRP)conjugated goat anti-mouse IgG secondary antibody (Sangon Biotech; 1:2000 dilution in PBS) was added and incubated for 1 h at 37℃. Colour development was initiated by adding TMB substrate (Beyotime), and the reaction was terminated with 1 M sulphuric acid. Optical density was measured at 450 nm using an Ensight Multimode Plate Reader (PerkinElmer). The endpoint titre was defined as the reciprocal of the highest serum dilution that yielded an absorbance value ≥2-fold above the blank control.

### Influenza virus microneutralization assay

The focus reduction neutralization test (FRNT) was employed to quantify the neutralizing antibodies present in immune sera. Briefly, MDCK cell monolayers were established by seeding cells into 96-well flat-bottom plates (Costar 3599, Corning) at a density of 3 × 10⁵ cells per well and culturing for 24 h under standard conditions (37°C, 5% CO₂). Sera obtained from immunized mice underwent pretreatment with receptor-destroying enzyme (RDE; Denka-Seiken 340122). This involved combining serum with RDE at a 1:3 ratio, incubating the mixture overnight (18 h) at 37°C, and subsequently heat-inactivating it at 56°C for 30 min. The treated sera were then subjected to three-fold serial dilutions in virus diluent (DMEM containing 25 mM HEPES and 2 μg/mL TPCK-trypsin), commencing at a 1:25 dilution. An aliquot of each serum dilution was combined with a challenge dose of 100 FFU of virus in MEM and allowed to interact for one hour at 37°C. These serum-virus complexes were then applied to the pre-formed MDCK monolayers. After a one-hour adsorption period, the inoculum was aspirated, and the cell layers were rinsed twice with phosphate-buffered saline (PBS). To restrict viral spread and facilitate focus formation, the cells were overlaid with 100 μL of a semi-solid medium consisting of MEM, 1% carboxymethyl cellulose (CMC), 1 μg/mL TPCK-trypsin, and 25 mM HEPES, followed by a 24-hour incubation at 37°C. Post-incubation, the cells were fixed with 4% paraformaldehyde. They were then permeabilized and blocked in a single step using a solution of 0.2% Triton X-100 and 1% bovine serum albumin (BSA). For immunostaining, the fixed cells were probed for one hour at 37°C with specific primary antibodies: either a mouse anti-nucleoprotein (NP) monoclonal antibody (4R5) or a rabbit anti-influenza B virus hemagglutinin (BV HA) antibody (Sino Biological). This was followed by a one-hour incubation with the corresponding horseradish peroxidase (HRP)-conjugated secondary antibodies: goat anti-mouse IgG or goat anti-rabbit IgG (Sangon Biotech; both at 1:2000 dilution). Viral foci were developed by applying a TMB-based substrate (TMB One Solution, YEASEN) for 10 min. The resulting foci were enumerated using an ELISpot reader (AT-Stop1100/CTL). The neutralization titre, reported as the FRNT₅₀, was calculated as the reciprocal of the maximum serum dilution that achieved a ≥ 50% reduction in focus count relative to the virus-only control wells [36].

### T-cell ELISpot assay

Antigen-specific T-cell responses were evaluated by enzyme-linked immunospot (ELISpot) assays measuring IFN-γ, IL-2, and TNF-α. Splenocytes were isolated from mice following either a single immunization or a prime-boost regimen, with assays conducted 12 days after the respective immunization. 96-well ELISpot plates (MabTech, Sweden) were coated overnight with capture antibodies against mouse IFN-γ (#33214HST-2), IL-2 (#3441-4HPW-2), and TNF-α (#3511-4HPW-2). Prior to cell plating, the coated plates were washed once with PBS and subsequently blocked for 30 min at room temperature with complete RPMI-1640 medium supplemented with 10% fetal bovine serum (FBS). Single-cell suspensions of splenocytes were seeded into the plates at a density of 2.5 × 10⁵ cells per well. The cells were stimulated with 2 μg/mL of peptide pools spanning the HA sequences of the relevant influenza strains. These pools included: W67-H1 (H1 strain), D6-H3 (H3 strain), and AUT21-BV (influenza B strain). All peptides were 15-mers with an 11-amino-acid overlap and synthesized to ≥90% purity (GenScript). Unstimulated wells served as negative controls. The plates were then incubated for 36 h at 37°C under 5% CO₂ to allow for cytokine secretion and capture. Following the incubation period, the cells were decanted, and the plates were subjected to five rigorous washes with 200 μL of PBS per well. The captured cytokines were detected by incubating the wells for 2 h at room temperature with biotinylated anti-cytokine antibodies (MabTech; 1 μg/mL in PBS containing 0.5% FBS). After another series of washes, streptavidin-conjugated horseradish peroxidase (HRP) was added to the wells and incubated for 1 h at room temperature. Spot development was achieved by adding a TMB substrate solution. The resulting cytokine spots were quantified using an automated ELISpot plate reader (PerkinElmer). The frequency of antigen-specific T cells was expressed as the number of spot-forming units (SFU) per million splenocytes after subtracting the background from unstimulated controls [36].

### Flow cytometry for antigen-specific T-cell responses

Antigen-specific T-cell responses were evaluated by intracellular cytokine staining following *in vitro* peptide restimulation. Splenocytes were isolated from mice following either a single immunization or a prime-boost regimen, and assays were performed 12 days after the respective immunization. Splenocytes (1 × 10^6^ cells per well) were cultured in RPMI-1640 medium supplemented with 10% fetal bovine serum (FBS) and stimulated with 5 μg/mL of peptide pools spanning the HA sequences of the relevant influenza strains, including W67-H1 (H1 strain), D6-H3 (H3 strain), and AUT21-BV (influenza B strain). All peptides were 15-mers with an 11-amino-acid overlap and synthesized to ≥90% purity (GenScript). Unstimulated cells served as negative controls, whereas Concanavalin A (10 μg/mL; InvivoGen, #inh-cona) was used as a positive control. GolgiPlug™ (BD Biosciences, #555029) and GolgiStop™ (BD Biosciences, #554724) were added at a 1:1,000 dilution, and the cells were incubated for 6 h at 37°C.

Following stimulation, cells were washed with PBS and surface-stained for 30 min at 4°C in the dark using anti-CD3e FITC (BD Biosciences, #553061), anti-CD4 PE-Cy7 (BD Biosciences, #552775), anti-CD8a BV510 (BD Biosciences, #563068), and Fixable Viability Stain 780 (BD Biosciences, #565388), diluted in MACS buffer (PBS containing 2% BSA and 1 mM EDTA). Cells were then fixed and permeabilized with Cytofix/Cytoperm solution (BD Biosciences, #554722) for 20 min at 4°C, followed by intracellular staining with anti-IFN-γ BV711 (BD Biosciences, #564336), anti-TNF-α BB700 (BD Biosciences, #566510), and anti-IL-2 PE (BD Biosciences, #554428), each diluted 1:100 in Perm/Wash buffer. After staining, cells were resuspended in MACS buffer and acquired on a NovoCyte Advanteon flow cytometer (Agilent Technologies). Data were analysed using Novo Express software, and antigen-specific responses were expressed after subtraction of background responses in unstimulated controls [36].

### Statistics

Data were analysed using GraphPad Prism version 10.2.3. The difference between two groups was calculated using a *t*-test. Statistically significant results were determined when *p* < 0.05, **p < 0.01, ***p < 0.001, ****p < 0.0001. Data are presented as the mean with standard deviations as indicated.

## Supplementary Material

Supplemental Material.docx
