# Supplementary material for: Enhanced immunogenicity and dose-sparing efficacy of self-amplifying RNA vaccines against seasonal influenza across subtypes
Source: Emerg Microbes Infect. 2026 May 4;15(1):2668752. doi: 10.1080/22221751.2026.2668752 (PMC13188553; doi:10.1080/22221751.2026.2668752)
Supplement: Supplemental Material.docx [file TEMI_A_2668752_SM0524.docx]

**Supplemental Material**

**Table S1. CAI and MFE-based optimization of PR8 (H1N1) HA sequence across**

**computational tools**

| **Software tool** | **CAI** | **ΔG(Kal/mol)** |
| --- | --- | --- |
| **Wild-type (WT)** | 0.7 | -419.1 |
| **GenSmart** | 0.93 | -562.5 |
| **Vecterbuilder** | 0.82 | -497.9 |
| **NovoPro** | 0.92 | -572 |
| **IDT** | 0.74 | -467.6 |
| **MFE** | 0.75 | -1103.8 |
| **CAI** | 1 | -657.2 |

**Table S2. Evaluation of CAI and MFE parameters in HA sequence optimization**

**across seasonal influenza subtypes (2023–2024)**

|  |  | **CAI** | **ΔG(Kal/mol)** |
| --- | --- | --- | --- |
| **W67** | WT | 0.71 | -409.10 |
|  | CAI | 0.99 | -443.90 |
|  | MFE | 0.75 | -942.9 |
| **D6** | WT | 0.71 | -427.10 |
|  | CAI | 0.99 | -598.60 |
|  | MFE | 0.74 | -944.5 |
| **AUT21** | WT | 0.7 | -336.30 |

CAI 1 -641.80

MFE

0.74

-

1038

1091

1092 **Table S3. Analysis of HA protein sequence similarity between vaccine strains and**

1093 **influenza A/B subtypes used in this study**

| **Subtype** | **Neutralization** |  | **Virus challenge** |  |
| --- | --- | --- | --- | --- |
|  | Strain | Sequence similarity | Strain | Sequence similarity |
| **H1** | A/Wisconsin/67/2022 | 100% | A/Puerto Rico/8/1934 | 89.58% |
|  | A/Michigan/45/2015 | 98.06% | A/California/07/2009 | 96.82% |
| **H3** | A/Darwin/6/2021 | 100% | A/Hong Kong/68 X-31 | 91.17% |
|  | A/Hong Kong/45/2019 | 97.35% |  |  |
| **BV** | B/Austria/1359417/2021 | 100% | B/Lee/1940 | 95.89% |
|  | B/Washington/02/2019 | 98.97% | B/Guangzhou/0215/2012 | 98.46% |

1094

1095
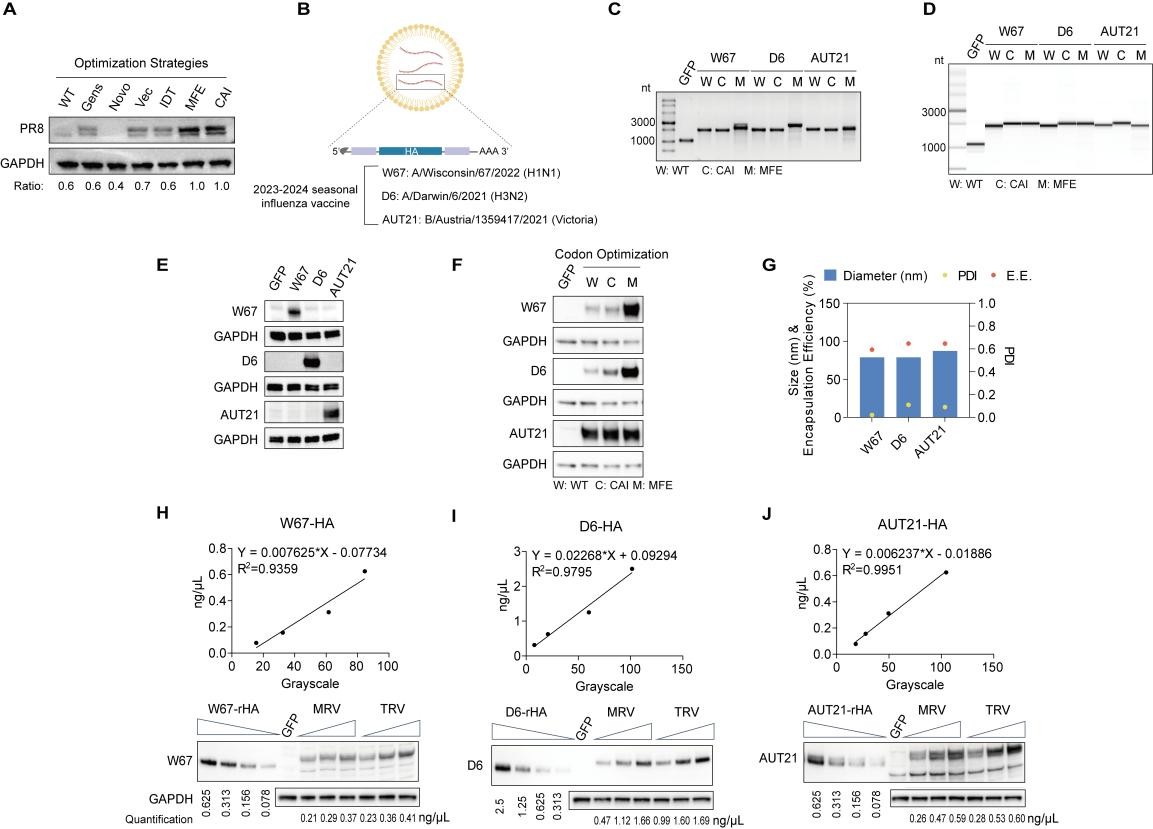


1096

1097 **Figure S1. HA expression analysis using different optimization approaches and** 1098 **RNA quality control.**

1. **(A)** Western blot analysis of HA expression from prototype strain PR8 (H1N1) with
2. different optimization strategies in BHK21 cells. **(B)** Schematic presentation of the
3. mRNA-LNP vaccine constructs encoding HA genes from WHO-recommended 2023-
4. 2024 seasonal influenza strains: A/Wisconsin/67/2022 (H1N1) (W67),
5. A/Darwin/6/2021 (H3N2) (D6), B/Austria/1359417/2021 (Victoria) (AUT21). **(C)**
6. Quality assessment of optimized influenza HA mRNAs by agarose gel
7. electrophoresis. **(D)** Quality assessment of optimized influenza HA mRNAs by
8. capillary electrophoresis. **(E)** WB analysis of antibody subtype specificity validated
9. by expression of MFE-optimized sequences in BHK21 cells. **(F)** Western blotting
10. analysis of HA protein expression in BHK21 cells. **(G)** Physicochemical
11. characterization of LNP-encapsulated HA. mRNA-LNP Z-average size and
12. polydispersity index (PDI) were measured by Dynamic Light Scattering (DLS), and 1111 encapsulation efficiency (E.E.) was determined using the RiboGreen RNA assay. **(H-**
13. **J)** Quantitative Western blot analysis of HA Protein Expression in BHK21 Cells.
14. BHK21 cells were transfected with varying doses (250, 500, and 1000 ng/μL) of 1114 either monovalent mRNA vaccine (MRV) or trivalent mRNA vaccine (TRV).
15. Recombinant proteins W67 (0.625, 0.313, 0.156, and 0.176 ng/μL), D6 (2.5, 0.625,
16. and 0.313 ng/μL), and AUT21 (0.625, 0.313, 0.156, and 0.176 ng/μL) were used as
17. quantitative standard. Protein expression levels were analyzed by grayscale value 1118 (G/P), with numerical values labeled below the corresponding bands (e.g., 0:23, 0:37

1119 for W67). GAPDH served as the loading controls.

1120

1121
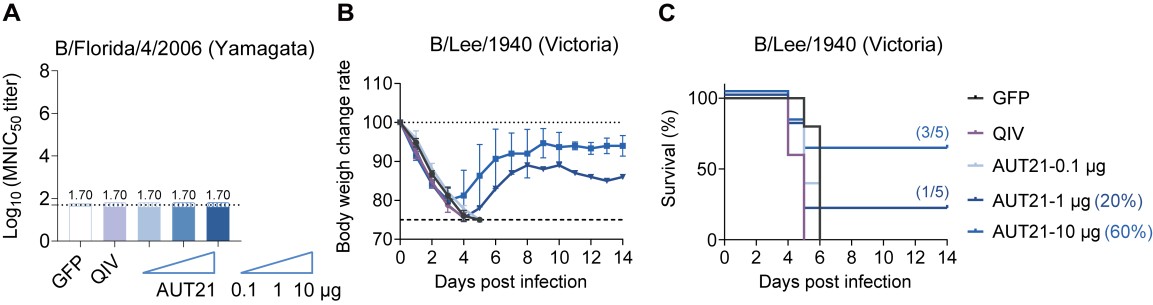


1122

1123 **Figure S2. Heterologous protection by monovalent mRNA-AUT21 vaccine.**

Mice were vaccinated (i.m.) with 0.1 μg, 1 μg or 10 µg mRNA vaccine using in a prime-boost schedule with an interval of 3 weeks. **(A)** Post-boost (week 6) serum neutralizing titers (NT50) against the B/Florida/4/2006 (Yamagata) virus. **(B)** At week 7, mice (n=5) were challenged with 5×LD_50_ of B-Lee40. Body weight changes. Data are presented as the mean (SD). **(C)** Survival curves. Data were analyzed using a log rank test.


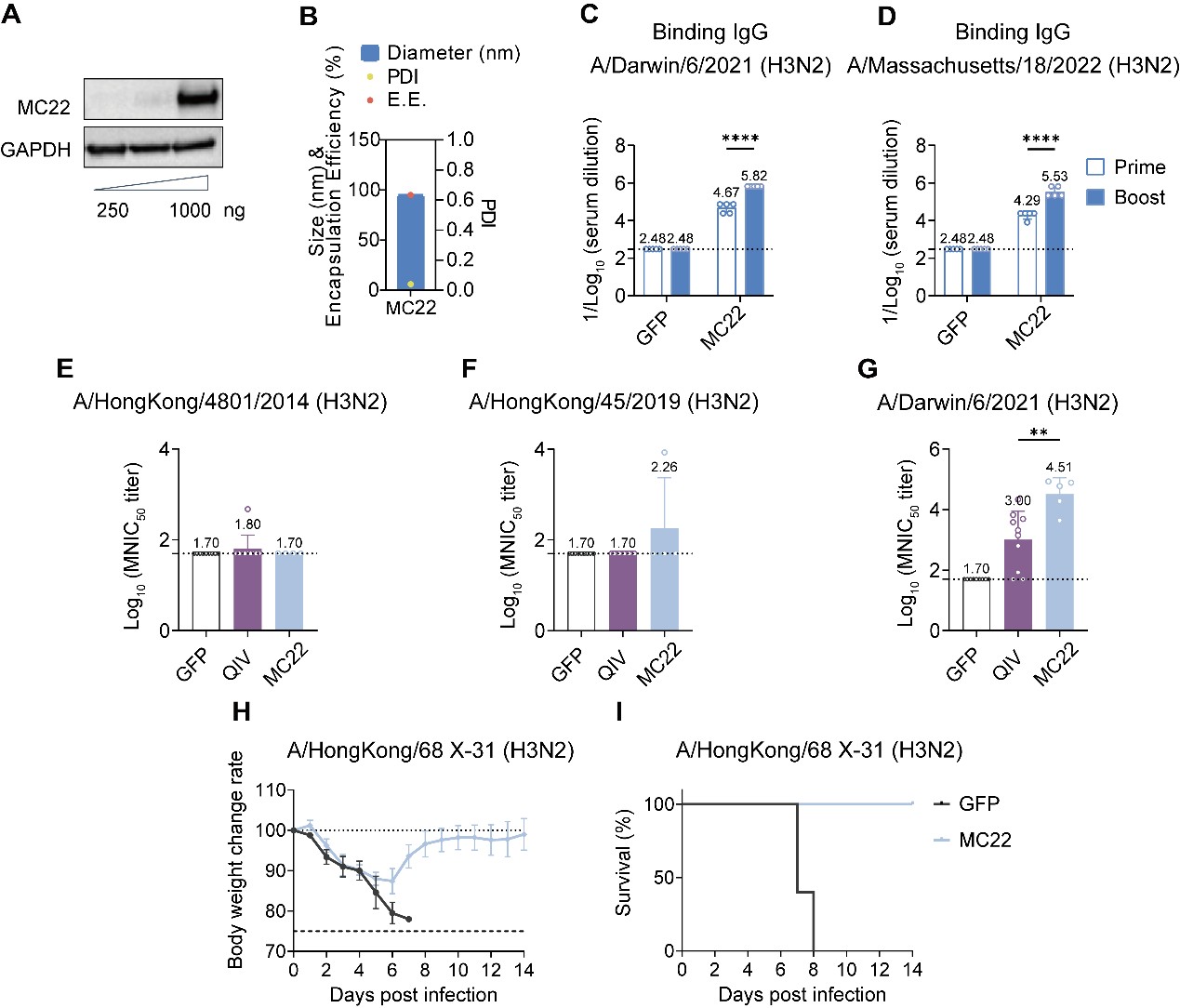


**Figure S3. Evaluation of prime-boost mRNA-MC22 vaccine immunization.** Mice received prime-boost intramuscular immunization with10 µg of SM102formulated mRNA-MC22. Sera were collected at week 3 and 6, with viral challenge performed at week 7. **(A)** Western blot detection of HA proteins in BHK21 cells transfected with mRNA-MC22. **(B)** Characterization of mRNA-MC22-LNPs. **(C-D)** HA-specific IgG ELISA (n=5). Serum binding antibodies against recombinant (C) D6 and (D) MC22 proteins were measured at week 3 or 6. **(E-G)** Neutralization assay (n=5). Post-boost (week 6) serum neutralizing titers (NT50) against (E)H3-HK14, (F)

H3-HK19, and (G) H3-D6 viruses. **(H-I)** Mice (n=5) were challenged with 5×LD_50_ of H3-HK68. (H) Body weight changes (mean (SD)). (I) Survival curves (analyzed by log-rank test). Data represent mean (SD); significance by two-tailed *t*-test (*p<0.05,

**p<0.01, ****p*<0.001, *****p*<0.0001).


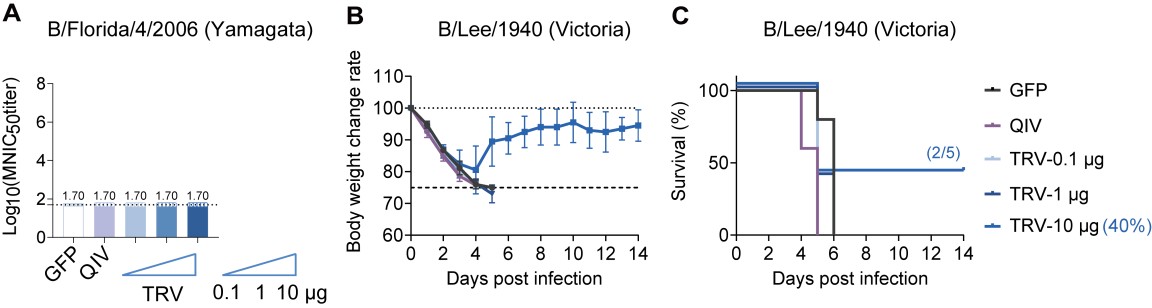


**Figure S4. Heterologous protection by trivalent mRNA vaccines.**

Mice were vaccinated (i.m.) with 0.1 μg, 1 μg or 10µg TRV using in a prime-boost schedule with an interval of 3 weeks. **(A)** Post-boost (week 6) serum neutralizing titers (NT50) against the B/Florida/4/2006 (Yamagata) virus. **(B)**At week 7, mice

(n=5) were challenged with 5×LD_50_ of B-Lee40 (Victoria). Body weight changes.

Data are presented as the mean (SD). **(C)** Survival curves. Data were analyzed using a log rank test.


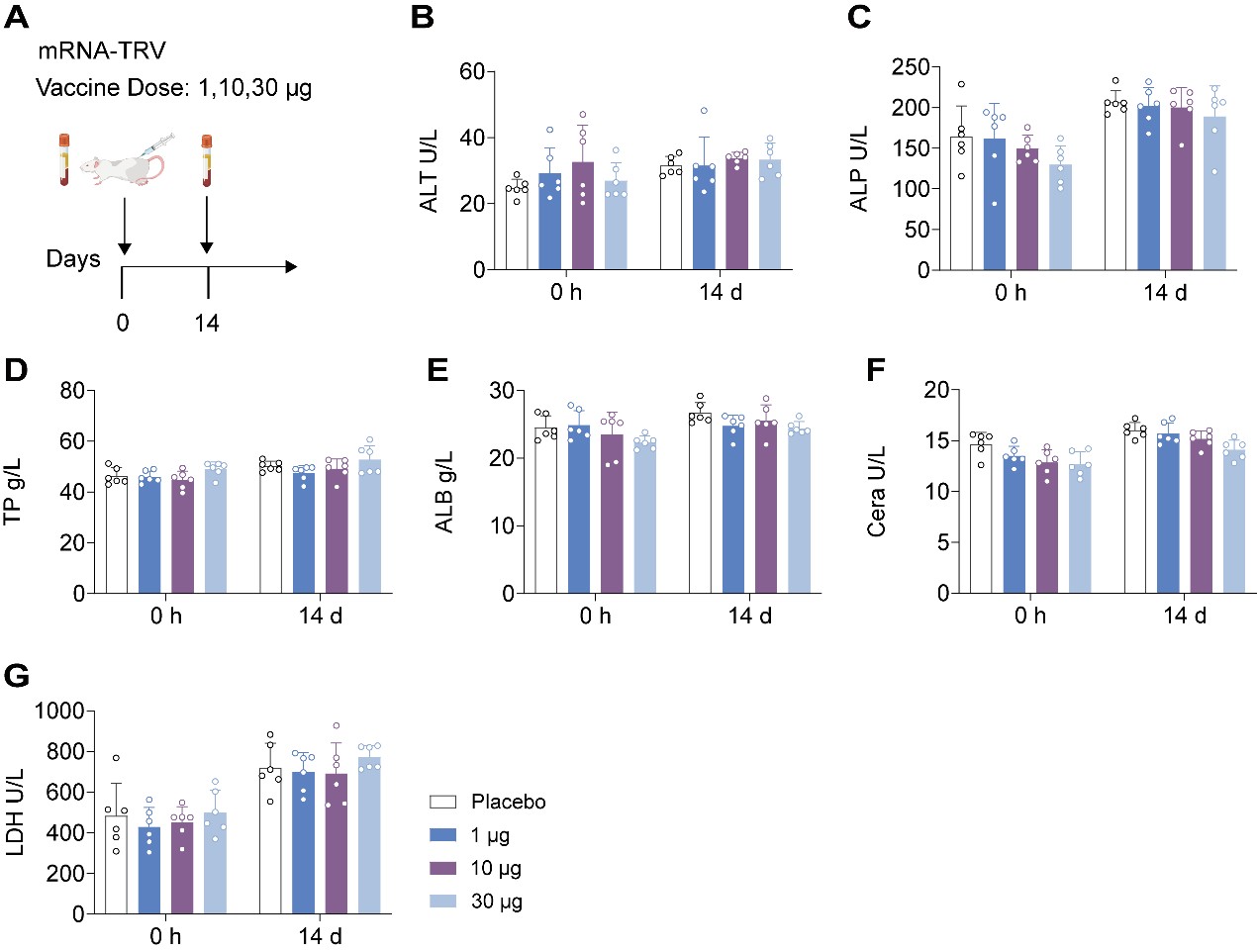


**Figure S5. Safety evaluation of trivalent mRNA vaccine in mice. (A)** Schematic illustration of the safety evaluation protocol. Mice were intramuscularly immunized with 1 μg, 10 μg, or 30 μg of mRNA-TRV, or placebo, and serum samples were collected at 0 h and 14 d after immunization. **(B-G)** Serum biochemical analysis of vaccinated mice. Levels of (B) alanine aminotransferase (ALT), (C) alkaline phosphatase (ALP), (D) total protein (TP), (E) albumin (ALB), (F) creatinine (Crea), and (G) lactate dehydrogenase (LDH) were measured at the indicated time points. Data represent mean (SD); significance by two-tailed t-test

(*p<0.05, **p<0.01, ***p<0.001, ****p<0.0001).


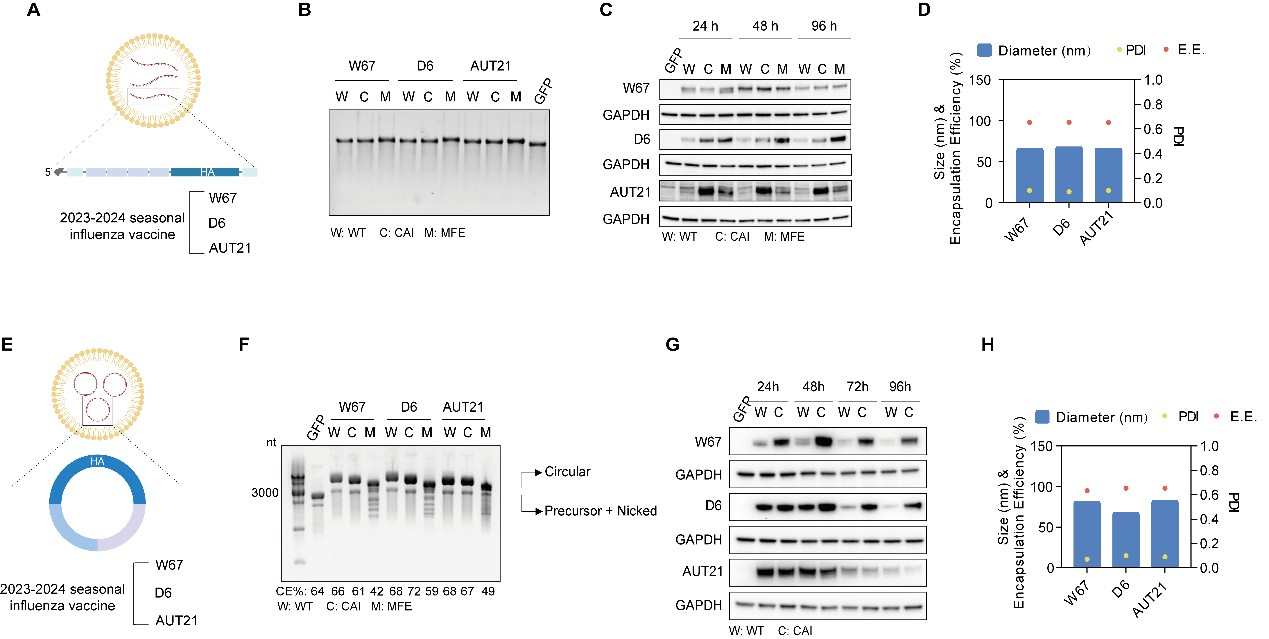


**Figure S6. Physicochemical properties of saRNA and circRNA *in vitro*.**

**(A)** Schematic presentation of saRNA constructs expressing HA antigens from 2023-2024 influenza vaccine strains: W67, D6, and AUT21. **(B)** Quality assessment of optimized saRNA by agarose gel electrophoresis. **(C)** Western blot analysis of HA protein expression in 293T cells transfected with saRNAs. **(D)** Characterization of saRNA-LNPs. **(E)** Schematic of circRNA constructs expressing HA antigens from 2023-2024 influenza strains: W67, D6, and AUT21. **(F)** Western blot detection of HA proteins in 293T cells transfected with circRNAs. **(G)** circRNA synthesis efficiency by E-gel electrophoresis. **(H)** Characterization of circRNA-LNPs.


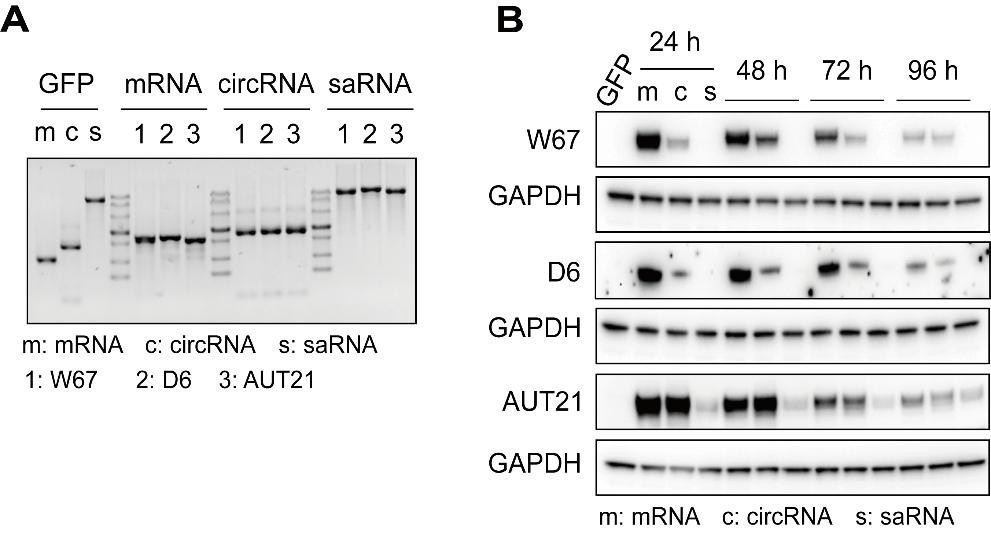


**Figure S7. Physicochemical properties of mRNA, saRNA and circRNA *in vitro*.**

1. Quality assessment of RNA produced by different platforms. **(B)** Western blotting analysis of different platforms HA protein expression in transfected 293T cells.


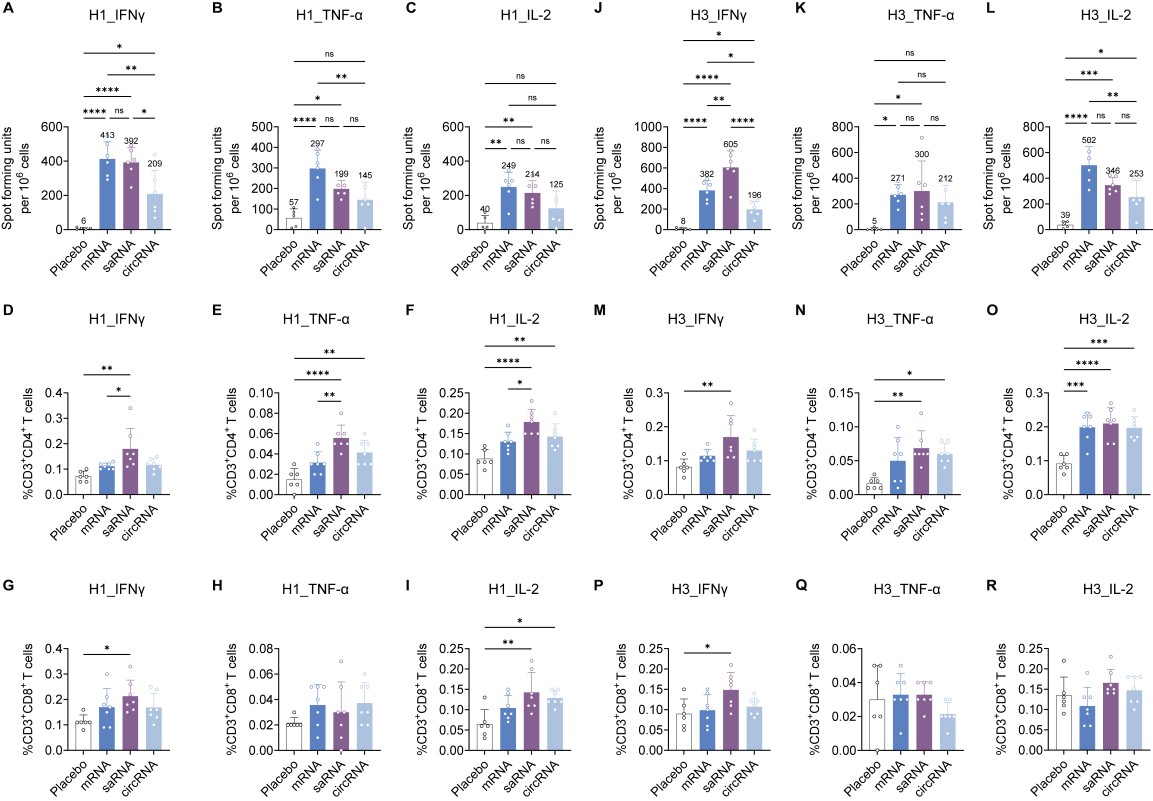


**Figure S8. Cellular immune responses against H1-W67 and H3-D6 induced by single-dose trivalent RNA vaccines.**

**(A-C)** ELISpot analysis of H1-W67-specific T cell responses following stimulation with H1-W67 peptide pool. Numbers of cytokine-secreting cells producing (A) IFN-γ, (B) TNF-α, and (C) IL-2 are shown. **(D-F)** Flow cytometric analysis of H1-W67specific CD4+ T cell responses. Frequencies of cytokine-positive CD3+CD4+ T cells cells producing (D) IFN-γ, (E) TNF-α, and (F) IL-2 are shown. **(G-I)** Flow cytometric analysis of H1-W67-specific CD8+ T cell responses. Frequencies of cytokine-positive CD3+CD8+ T cells producing (G) IFN-γ, (H) TNF-α, and (I) IL-2 are shown. **(J-L)**

ELISpot analysis of H3-D6-specific T cell responses following stimulation with H3D6 peptide pool. Numbers of cytokine-secreting cells producing (J) IFN-γ, (K) TNFα, and (L) IL-2 are shown. **(M-O)** Flow cytometric analysis of H3-D6-specific CD4+

T cell responses. Frequencies of cytokine-positive CD3+CD4+ T cells producing (M)

IFN-γ, (N) TNF-α, and (O) IL-2 are shown. **(P-R)** Flow cytometric analysis of H3D6-specific CD8+ T cell responses. Frequencies of cytokine-positive CD3+CD8+ T cells producing (P) IFN-γ, (Q) TNF-α, and (R) IL-2 are shown. Data represent mean (SD); significance by two-tailed t-test (*p<0.05, **p<0.01, ***p<0.001,

****p<0.0001).


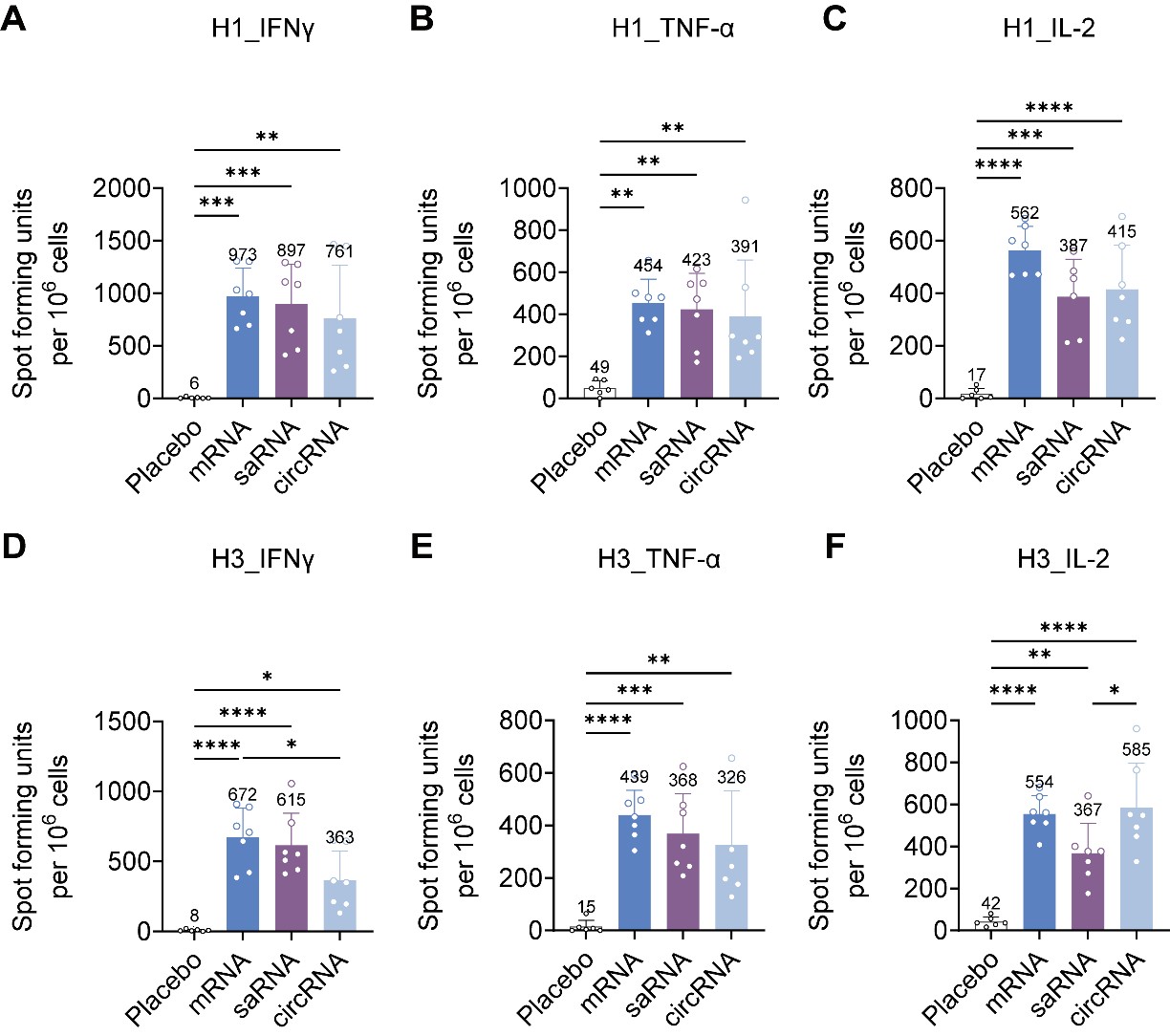


**Figure S9. ELISpot analysis of H1-W67- and H3-D6-specific T cell responses following prime-boost trivalent RNA vaccination.**

**(A-C)** ELISpot analysis of H1-W67-specific T cell responses following stimulation with H1-W67 peptide pool. Numbers of cytokine-secreting cells producing (A) IFN-γ, (B) TNF-α, and (C) IL-2 are shown. **(D-F)** ELISpot analysis of H3-D6-specific T cell responses following stimulation with H3-D6 peptide pool. Numbers of cytokinesecreting cells producing (D) IFN-γ, (E) TNF-α, and (F) IL-2 are shown. Data represent mean (SD); significance by two-tailed t-test (*p<0.05, **p<0.01,

***p<0.001, ****p<0.0001).


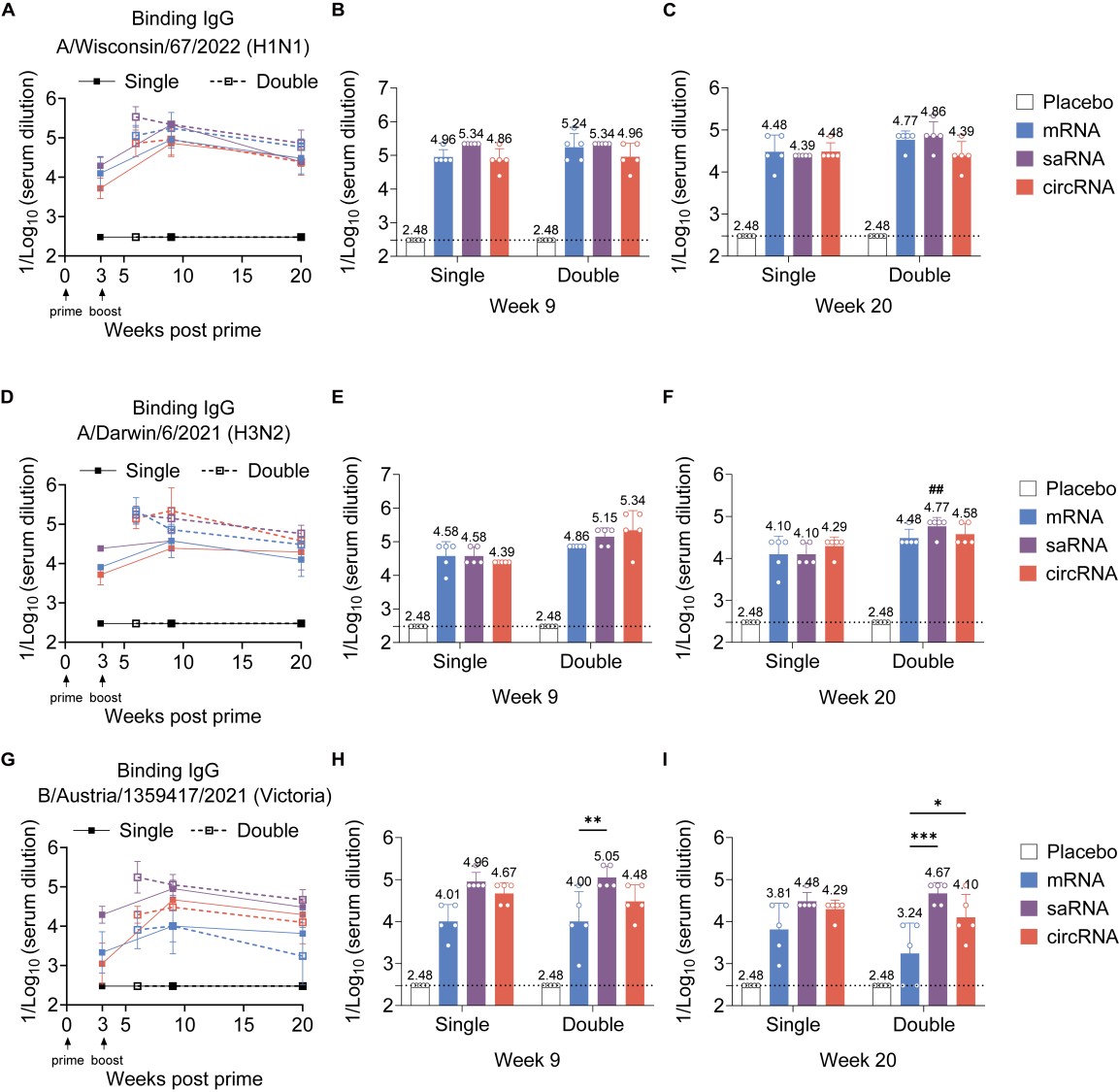


**Figure S10. Detection of long-term antibodies following a 1 μg dose of mRNA, saRNA, and circRNA vaccines.**

Serum samples were collected from mice at weeks 3, 6, 9 and 20 after the initial immunization. A comparison was made between single-dose and double-dose immunization strategies, with administered doses of HA-specific IgG ELISA (n=5).

Serum binding antibodies against recombinant **(A-C)** W67-H1, **(D-F)** D6-H3, and (GI) AUT21-BV proteins were measured. Data represent mean (SD); significance by two-tailed t-test (*p<0.05, **p<0.01, ***p<0.001, ****p<0.0001; single vs. double

#p<0.05, ##p<0.01, ###p<0.001, ####p<0.0001).


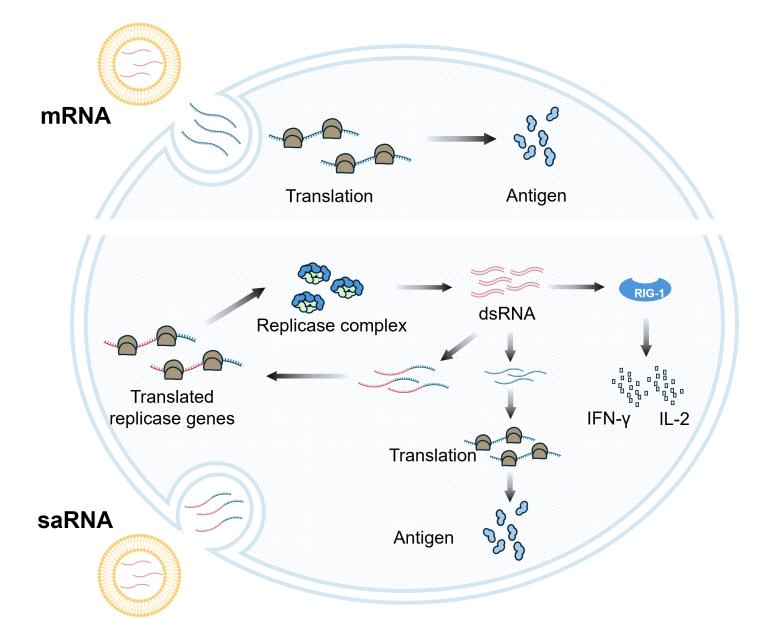


**Figure S12. Schematic comparison of antigen expression mechanisms between conventional mRNA and saRNA vaccines.**

After delivery into host cells, conventional mRNA is translated directly into antigen, resulting in relatively limited antigen expression. By contrast, saRNA encodes viral replicase proteins that mediate intracellular RNA amplification, thereby increasing antigen-encoding RNA levels and prolonging or enhancing antigen production. This replication process generates double-stranded RNA (dsRNA) intermediates, which can be sensed by pattern recognition receptors such as RIG-I and MDA5, thereby triggering innate immune responses. These properties may underlie the enhanced immunogenicity and low-dose efficacy commonly associated with saRNA vaccines.
